# Identification of genome-wide SNP-SNP interactions associated with important traits in chicken

**DOI:** 10.1186/s12864-017-4252-y

**Published:** 2017-11-21

**Authors:** Hui Zhang, Jia-Qiang Yu, Li-Li Yang, Luke M. Kramer, Xin-Yang Zhang, Wei Na, James M. Reecy, Hui Li

**Affiliations:** 1 Key Laboratory of Chicken Genetics and Breeding, Ministry of Agriculture, Harbin, 150030 People’s Republic of China; 2 Key Laboratory of Animal Genetics, Breeding and Reproduction, Education Department of Heilongjiang Province, Harbin, 150030 People’s Republic of China; 3 College of Animal Science and Technology, Northeast Agricultural University, Harbin, 150030 People’s Republic of China; 4 Department of Animal Science, Iowa State University, 2255 Kildee Hall, Ames, IA 50011 USA

**Keywords:** Carcass and growth traits, Testis, Epistasis, SNP-SNP interaction, Chicken

## Abstract

**Background:**

In addition to additive genetic effects, epistatic interactions can play key roles in the control of phenotypic variation of traits of interest. In the current study, 475 male birds from lean and fat chicken lines were utilized as a resource population to detect significant epistatic effects associated with growth and carcass traits.

**Results:**

A total of 421 significant epistatic effects were associated with testis weight (TeW), from which 11 sub-networks (Sub-network1 to Sub-network11) were constructed. In Sub-network1, which was the biggest network, there was an interaction between GGA21 and GGAZ. Three genes on GGA21 (*SDHB*, *PARK7* and *VAMP3*) and nine genes (*AGTPBP1*, *CAMK4*, *CDC14B*, *FANCC*, *FBP1*, *GNAQ*, *PTCH1*, *ROR2* and *STARD4*) on GGAZ that might be potentially important candidate genes for testis growth and development were detected based on the annotated gene function. In Sub-network2, there was a SNP on GGA19 that interacted with 8 SNPs located on GGA10. The SNP (Gga_rs15834332) on GGA19 was located between C-C motif chemokine ligand 5 (*CCL5*) and *MIR142*. There were 32 Refgenes on GGA10, including *TCF12* which is predicted to be a target gene of miR-142-5p. We hypothesize that miR-142-5p and *TCF12* may interact with one another to regulate testis growth and development. Two genes (*CDH12* and *WNT8A*) in the same cadherin signaling pathway were implicated as potentially important genes in the control of metatarsus circumference (MeC). There were no significant epistatic effects identified for the other carcass and growth traits, e.g. heart weight (HW), liver weight (LW), spleen weight (SW), muscular and glandular stomach weight (MGSW), carcass weight (CW), body weight (BW1, BW3, BW5, BW7), chest width (ChWi), metatarsus length (MeL).

**Conclusions:**

The results of the current study are helpful to better understand the genetic basis of carcass and growth traits, especially for testis growth and development in broilers.

**Electronic supplementary material:**

The online version of this article (10.1186/s12864-017-4252-y) contains supplementary material, which is available to authorized users.

## Background

Epistasis arises due to interactions, either between single nucleotide polymorphisms (SNPs), genes or quantitative trait loci (QTLs), which result in non-linear effects that control variation in phenotypes. Epistasis can have a large influence on phenotypic variation of traits such as starvation resistance, startle response, and chill coma recovery in Drosophila [1]. Identification of epistatic effects associated with quantitative traits will help us to better understand the genetic architecture that underlies complex variation of phenotypes for both humans and animals [1, 2]. Therefore, more and more attention has been placed on epistasis, which has resulted in some valuable insights [3–8].

With the advent of SNP arrays and genomic re-sequencing, it is relatively easy to genotype a wide array of individuals. As a result, many genome–wide association studies (GWAS) have been carried out in the past several years. Most of these studies have focused on single locus additive genetic tests. However, this is not that only type of genetic association. A genome wide SNP-SNP interaction analysis should provide new insights into the genetic architecture that underlies variation in complex traits.

Recently, genome wide SNP-SNP interaction analysis have been conducted in humans [4, 9] and domestic livestock species [10–12]. In chicken (*Gallus gallus*), it has been suggested that epistatic interactions between genes (or QTLs) are important for variation in quantitative traits [8, 13, 14]. However, the study of epistatic interactions at the whole genome level have been limited [15].

Two Northeast Agricultural University broiler lines that have been divergently selected for abdominal fat content (named as NEAUHLF) for more than 10 years were used in the current study. Previously, we reported that 52 pairs of SNPs had significant epistatic interactions that were associated with abdominal fat weight [15]. In the current study, the significant epistatic interactions for carcass and growth traits were identified. The results of this study may provide some helpful information to better understand the genetic basis of carcass and growth traits in broilers.

## Methods

### Experimental populations

Two Northeast Agricultural University broiler lines that have been divergently selected for abdominal fat content (NEAUHLF) were used to identify epistatic interactions. The NEAUHLF lines have been selected since 1996 using abdominal fat percentage (AFP = abdominal fat weight/body weight at 7 weeks of age) and plasma very low-density lipoprotein (VLDL) concentration as selection criteria. The G0 generation of NEAUHLF came from the same grandsire line, which originated from the Arbor Acres broiler, which was then divided into two lines according to their plasma VLDL concentration at 7 weeks of age. The G0 birds were mated (one sire: four dams) to produce 25 half-sib families for each line, with an average of 70 G1 offspring per family in two hatches. From G1 to G11, the birds of each line were raised in two hatches with five birds per cage. Plasma VLDL concentrations were measured for all male birds, which had free access to feed and water at 7 weeks, and the AFP of the male birds in the first hatch was measured after slaughter at 7 weeks. Sib birds from the families with lower (lean line) or higher (fat line) AFP than the average value for the population were selected as candidates for breeding, considering the plasma VLDL concentration and the body weights of male birds in the second hatch and the egg production of female birds in both hatches. These birds were kept under the same environmental conditions and had free access to feed and water. Commercial corn-soybean-based diets that met all National Research Council (NRC) requirements were provided. From hatch to 3 weeks of age, the birds received a starter feed (3,000kal ME = kg and 210 g = kg CP) and from 4 weeks of age to slaughter the birds were fed a grower diet (3100 kal ME = kg and 190 g = kg CP). The birds used in the current study included 475 male individuals from the 11th generation of NEAUHLF [15]. The birds were weighed at 0, 1, 3, 5 and 7 weeks of age (BW0, BW1, BW3, BW5 and BW7). At 7 weeks of age, the metatarsus length (MeL), metatarsus circumference (MeC) and chest width (ChWi) were measured prior to slaughter as described previously [16]. Carcass weight (CW), testis weight (TeW), heart weight (HW), liver weight (LW), spleen weight (SW), muscular and glandular stomach weight (MGSW) were obtained after the birds were slaughtered.

### SNP genotyping

Genotyping was carried out using the Illumina Inc. (San Diego, CA, USA) chicken 60 K SNP chip, which contained 57,636 SNPs. After quality control, 48,824 SNPs in 475 individuals were used in the epistatic interaction analyses. The quality control of the SNP genotypes was described previously by Zhang et al. [17].

### Genome-wide Pairwise interaction analysis

The EPISNP3 module in epiSNP_v4.2_Windows software package was used to identify significant epistatic effects [18]. The statistical model used to test for epistatic effects associated with carcass and growth traits was as follows: y = Xg + Zb + e, where y is the column vector of phenotypic values, g is the effects of SNP genotypes, X is the design matrix of g, b is the fixed effects of Line and BW7 (or BW0), Z is the model matrix of b, and e is the random error.

The *P*-values of the epistatic effects were Bonferroni corrected for multiple testing (5.96 × 10^9^ independent tests, with a significance threshold of *P* < 0.05), which resulted in *P* < 8.39 × 10^−12^ as a significance threshold. Significant interactions, including additive by additive (AA), additive by dominance (AD) or dominance by additive (DA) and dominance by dominance (DD), between two SNPs on the same chromosome were deleted because these interactions may potentially be markers for a haplotype effect that contains a single QTL [12]. The remaining significant SNP interactions were further filtered with the criterion that only those interaction with at least 10 animals in every genotype combination were considered [12], which is roughly equivalent to a 15% minor allele frequency for each variant in an additive by additive epistatic interaction.

### SNP-SNP network

The figures that illustrate the SNP-SNP networks with the significant epistatic effects for carcass and growth traits were drawn using the epiNet option within the epiSNP_v4.2_Windows software package [18].

### Linkage disequilibrium (LD) analysis

The linkage disequilibrium (LD) between SNPs was calculated using Haploview software (version 4.2). The solid spine method within the package was used to define the LD block.

### Annotation of SNP-SNP network

Genes within 1 Mb (upstream and downstream) of the SNPs that had significant interactions with another SNP for carcass and growth traits were retrieved from UCSC (https://genome.ucsc.edu/) (Galgal4). Functional annotation of genes was performed using DAVID bioinformatics resources 6.8 (http://david.abcc.ncifcrf.gov/summary.jsp) for Gene Ontology (GO) terms and Kyoto Encyclopedia of Genes and Genomes (KEGG) pathway analysis. Statistical significance was set at the nominal *P*-value < 0.05.

## Results

### Phenotypic and SNPs information

Phenotypic summary statistics for carcass and growth traits are shown in Table 1 and the phenotypic distributions for the traits in the lean and fat lines respectively are shown in Additional file 1: Figure S1. There were extremely significant differences (*P* < 0.01) in Chwi, MeL, MeC, LW, SW, TeW and significant difference (*P* < 0.05) in HW between the lean and fat lines. After quality control, 48,824 SNPs were utilized for epistatic interaction analyses (Table 2). These SNPs were distributed on 28 autosomes, Z chromosome, two linkage groups, and SNPs not assigned to any chromosomes in chickens. These markers covered about 1027.01 Mb of the chicken genome, with an average SNP density of 16.08 kb/SNP.

**Table 1:** The Mean ± Standard deviation (SD) of the carcass and growth traits in lean and fat lines, respectively, and in the combined population

Traits	Combined population (475 birds)	Lean line (203 birds)	Fat line (272 birds)
BW1 (g)	121.97 ± 12.34	121.05 ± 12.80	122.68 ± 11.95
BW3 (g)	615.22 ± 65.97	617.35 ± 71.98	613.65 ± 61.23
BW5 (g)	1491.19 ± 142.53	1487.53 ± 159.13	1493.91 ± 129.10
BW7 (g)	2400.97 ± 221.65	2419.53 ± 246.45	2387.11 ± 200.51
ChWi (cm)	9.23 ± 0.74	9.54 ± 0.70^A^	9.00 ± 0.68^B^
MeL (cm)	9.25 ± 0.46	9.56 ± 0.37^A^	9.02 ± 0.38^B^
MeC (cm)	5.10 ± 0.39	5.46 ± 0.27^A^	4.84 ± 0.20^B^
CW (g)	2170.03 ± 203.31	2164.95 ± 225.19	2173.84 ± 185.59
LW (g)	57.54 ± 9.08	55.35 ± 8.44^B^	59.18 ± 9.21^A^
HW (g)	10.68 ± 1.72	10.86 ± 1.78^A^	10.54 ± 1.67^a^
SW (g)	3.25 ± 1.05	2.88 ± 0.86^B^	3.53 ± 1.09^A^
MGSW (g)	31.10 ± 5.57	31.46 ± 6.07	30.83 ± 5.15
TeW (g)	1.03 ± 0.85	1.39 ± 1.03^A^	0.77 ± 0.55^B^

Note: Different letters indicate significant differences between the lean and fat lines. Uppercase (*P* < 0.01) and lowercase (*P* < 0.05) letters indicate significant differences

**Table 2:** Summary information of the genome-wide SNP markers

GGA^1^	SNPs number	GGA length (Mb)	Mean distance (kb)
1	7538	200.95	26.66
2	5652	154.79	27.39
3	4322	113.65	26.30
4	3518	94.16	26.77
5	2295	62.23	27.11
6	1814	35.84	19.76
7	1907	38.17	20.01
8	1486	30.62	20.61
9	1240	24.02	19.37
10	1379	22.42	16.26
11	1312	21.87	16.67
12	1425	20.46	14.36
13	1204	18.32	15.21
14	1062	15.76	14.84
15	1082	12.93	11.95
16	16	0.42	26.12
17	922	10.61	11.51
18	917	10.89	11.87
19	880	9.90	11.25
20	1574	13.92	8.84
21	796	6.95	8.73
22	327	3.89	11.90
23	643	6.02	9.37
24	758	6.37	8.40
25	181	2.02	11.17
26	670	5.03	7.51
27	506	4.84	9.56
28	607	4.47	7.37
LGE22C19W28_E50C23	115	0.88	7.67
LEG64	3	0.02	6.80
Z	2001	74.59	37.28
UN^a^	672	/	/
Total/Mean value	48,824	1027.01	16.08

^a^These SNPs were not assigned to any chromosomes

^1^GGA is an abbreviation for Gallus gallus

### Epistatic analysis of carcass trait

The pairwise interaction effects between every two SNPs across the whole chicken genome for TeW were calculated using EPISNP3 [18]. After filtering, 421 pairs of SNPs were significantly associated with testis weight (*P* < 8.39 × 10^−12^). Of these 421 significant SNP by SNP interactions, 403 (95.72%) exhibited an additive by additive interaction, 18 (4.28%) exhibited an additive by dominance (or dominance by additive) interaction, and no dominance by dominance interactions were detected (Additional file 2: Table S1). The most significant additive by additive effect detected occurred between GGA3 (GGaluGA22768) and GGA10 (Gga_rs14722408). The phenotypic distributions of the four different genotype classes of this additive by additive effect are showed in Additional file 3: Figure S2.

**Fig. 1:**
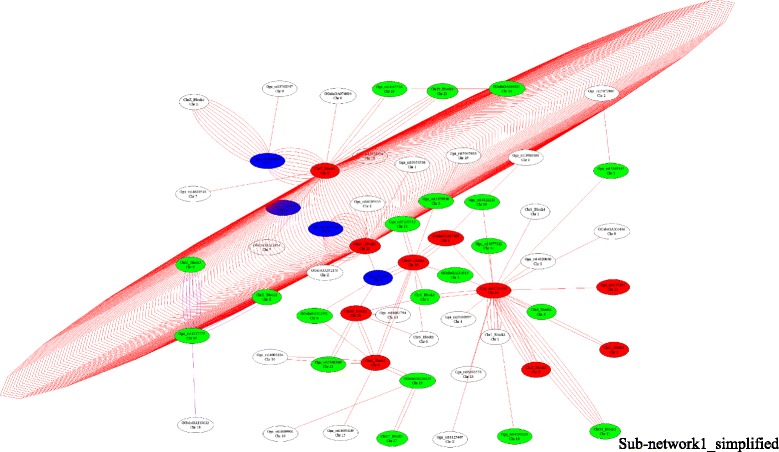
A simplified network of Sub-network1 using the LD information. The blocks are from the LD information in Additional file 3: Figure S2 and the lines between two nodes depicts an interaction between these two nodes. The colors of the nodes represent the *P*-value of an interaction (*P* < 1.0 × 10^−16^ = red; *P* < 1.0 × 10^−15^ = blue; *P* < 1.0 × 10^−14^ = green; *P* < 1.0 × 10^−13^ = white). The color of the edge indicates the type of epistatic effect (AA = red; AD = purple; DA = blue; DD = green)

To investigate the complex mechanism of epistatic effects on TeW, networks were constructed using the 421 significant SNP by SNP interactions. The epistatic interaction sub-networks that contained more than three nodes are shown in Additional file 4: Figure S3. Eleven sub-networks were detected (Additional file 4: Figure S3). Sub-network1 was the biggest and contained 372 pairs of SNP by SNP interaction effects. Based on LD information, a simpler sub-network, which was derived from Sub-network1, was obtained, in which numerous SNPs are represented by a single LD block (Fig. 1, Additional file 5: Figure S4). The blocks in the simpler sub-network represented the SNPs that contained in the LD blocks. Two hundred and fifty-five of the interactions in Sub-network1 were between GGA21 and GGAZ, which indicated an interaction between the two chromosomes. The 255 interactions detected between GGA21 and GGAZ involved 24 SNPs on GGA21 and 19 SNPs on GGAZ, which spanned 572 kb (from 76,023 bp to 647,587 bp) and 9.5 Mb (from 37,246,321 bp to 46,745,968 bp), respectively. There were 13 Refgenes in the 572 kb region on GGA21 and 41 Refgenes in the 9.5 Mb region on GGAZ (Table 3). Six GO terms, including protein-arginine deiminase activity, protein citrullination, positive regulation of collateral sprouting, cytoplasm, cell fate determination and protein autophosphorylation, were significantly (*P* < 0.05) enriched. No significant KEGG pathways were detected. Three genes on GGA21 (*SDHB*, *PARK7* and *VAMP3*) and nine genes (*AGTPBP1*, *CAMK4*, *CDC14B*, *FANCC*, *FBP1*, *GNAQ*, *PTCH1*, *ROR2* and *STARD4*) on GGAZ might be important for testis growth and development based on their basic functions.

**Table 3:** The SNPs on GGAZ interacted with the SNPs on GGA21, and the Refgenes in the two important regions

Chr	Position	Locus	RS#	Refgenes
Z	37,246,321 37,440,770 37,562,582 38,066,346 38,424,448 38,545,044 38,795,339 39,120,198 39,189,946 39,223,668 39,256,770 39,415,378 40,393,058 40,411,585 40,514,085 41,304,561 45,300,030 45,695,927 46,745,968	Gga_rs14765324 Gga_rs16768474 Gga_rs14765605 Gga_rs16768723 Gga_rs14766107 GGaluGA351567 Gga_rs16047676 Gga_rs16781643 Gga_rs14745723 Gga_rs16131986 Gga_rs14787078 Gga_rs16781713 Gga_rs14787751 Gga_rs16782083 Gga_rs16754179 Gga_rs16132921 Gga_rs14015526 GGaluGA352176 Gga_rs14016510	rs14765324 rs16768474 rs14765605 rs16768723 rs14766107 rs317567123 rs16047676 rs16781643 rs14745723 rs16131986 rs14787078 rs16781713 rs14787751 rs16782083 rs16754179 rs16132921 rs14015526 rs316100592 rs14016510	*VPS13A*, *AGTPBP1*, *AUH*, *CAMK4*, *CDC14B*, *CDC42SE2*, *CFC1B*, *CKS2*, *CTSL2*, *DAPK1*, *FANCC*, *FBP1*, *GADD45B*, *GNAQ*, *HABP4*, *HINT1*, *ISCA1*, *LOC427470*, *LOC770548*, *MIR1456*, *MIR23B*, *MIR24*-2, *MIR27B*, *MIR7-1*, *MIR7439*, *NAA35*, *NFIL3*, *NREP*, *NTRK2*, *PTCH1*, *REEP5*, *RMI1*, *ROR2*, *SEMA4D*, *SLC25A46*, *SPINZ*, *STARD4*, *SYK*, *TLE4*, *WDR36*, *ZCCHC6*
21	76,023 87,719 113,769 125,790 140,660 145,617 153,135 165,390 197,067 219,311 277,332 291,087 297,203 299,152 304,979 320,390 332,352 332,687 362,742 402,941 423,299 493,436 631,537 647,587	Gga_rs15179992 GGaluGA181809 Gga_rs15179999 Gga_rs10732124 Gga_rs15180005 Gga_rs15180007 GGaluGA181823 Gga_rs16176404 Gga_rs15180023 Gga_rs15180012 Gga_rs15180032 Gga_rs15180041 Gga_rs16176409 Gga_rs16176412 GGaluGA181852 Gga_rs16176425 GGaluGA181865 GGaluGA181868 GGaluGA181877 Gga_rs14281175 Gga_rs13602346 Gga_rs14281291 Gga_rs16176824 GGaluGA182048	rs15179992 rs312621287 rs15179999 rs10732124 rs15180005 rs15180007 rs315641745 rs16176404 rs15180023 rs15180012 rs15180032 rs15180041 rs16176409 rs16176412 rs314825899 rs16176425 rs314965954 rs313888034 rs316891294 rs14281175 rs13602346 rs14281291 rs16176824 rs312963281	*PADI3*, *PADI1*, *PADI2*, *SDHB*, *MRPS16*, *PARK7*, *UTS2*, *PER3*, *VAMP3*, *PHF13*, *ZBTB48*, *ICMT*, *RPL22*

**Fig. 2:**
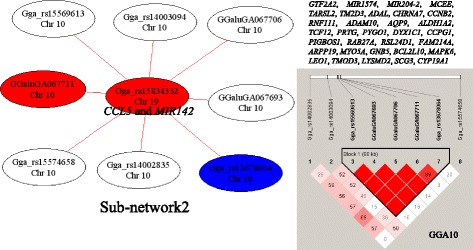
Sub-network2 for testis weight (TeW) and the LD information. The color of the node represents the *P*-value of an interaction (*P* < 1.0 × 10^−16^ = red; *P* < 1.0 × 10^−15^ = blue; *P* < 1.0 × 10^−14^ = green; *P* < 1.0 × 10^−13^ = white). The color of the edge indicates the type of epistatic effect (AA = red; AD = purple; DA = blue; DD = green). The genes located in the 4.3 Mb regions of GGA10 were listed

Sub-networks 2, 3, 4, 5, 8 and 11 each had several SNPs in the same LD block on one chromosome, which interacted with a single SNP on another chromosome. For example, in Sub-network2 eight SNPs on GGA10 interacted with the SNP (Gga_rs15834332) on GGA19 (Fig. 2). The eight SNPs on GGA10 were spread across a 4.3 Mb region that contained 32 chicken Refgenes (Fig. 2). The SNP Gga_rs15834332 on GGA19 was located between two genes, C-C motif chemokine ligand 5 (*CCL5*) and *MIR142*, which is related to two miRNAs, miR-142-5p and miR-142-3p. Interestingly, five genes in the 4.3 Mb region on GGA10 are predicted to be target genes of miR-142-5p and miR-142-3p (Table 4). Among these five target genes, transcription factor 12 (*TCF12*) was the only gene that was predicted to be the target gene of miR-142-5p by three different packages, including TargetScan (http://www.targetscan.org), miRDB (http://mirdb.org) and PicTar (http://pictar.mdc-berlin.de). Unfortunately, it was impossible to predict which genes in the other regions of the genome may be good candidates to control testis growth and development.

**Table 4:** Target genes of miR-142-5p and miR-142-3p in the 4.3 Mb region on GGA10 in Sub-network2 for TeW predicted by three packages online

Gene symbol	Description	Position (Mb)	MiRNA	Packages
*RNF111*	Ring Finger Protein 111	6.32–6.36	miR-142-3p	Targetscan
*TCF12*	transcription factor 12	6.90–7.00	miR-142-5p	MIRDB, Targetscan PicTar
*ARPP19*	cAMP–regulated phosphoprotein, 19 kDa	8.23–8.24	miR-142-5p	Targentscan
*MYO5A*	myosin VA (heavy chain 12, myoxin)	8.24–8.33	miR-142-3p	Targentscan
*MAPK6*	Mitogen–Activated Protein Kinase 6	8.42– 8.45	miR-142-5p	Targentscan

For CW, HW, LW, SW and MGSW, no significant epistatic interactions were detected.

### Epistatic analysis of growth trait

**Fig. 3:**
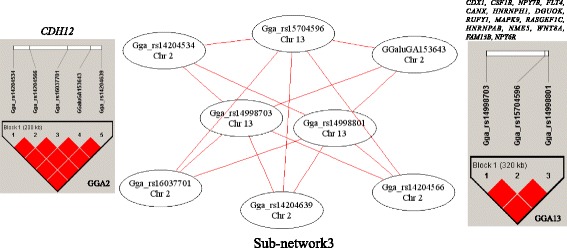
Epistatic network among SNPs with significant epistatic effect on metatarsus circumference (MeC). A node represents a SNP. The chromosome in which the SNP is located is shown in the circle. A pair of SNPs connected by an edge had a significant interaction. The color of a node represent the P-value of the interaction (*P* < 1.0 × 10^−12^ = red; *P* < 1.0 × 10^−11^ = blue; *P* < 1.0 × 10^−10^ = green; *P* < 1.0 × 10^−9^ = white). The color of the edge indicates the type of epistatic effect (AA = red; AD = purple; DA = blue; DD = green). Genes in the LD blocks were listed

For BW1, BW3, BW5, BW7, ChWi and MeL, no significant epistatic interactions were detected. Fifteen pairs of SNPs with significant interaction effects on MeC were detected (Table 5). These 15 interactions were all additive by additive interactions, which implicated an interaction between GGA2 and GGA13 (Table 5). There was a single network that contained all 15 interactions (Fig. 3). The fifteen interactions occurred between five SNPs in a single LD block on GGA2, and three SNPs in a single LD block on GGA13 (Fig. 3). The genes inside the two LD blocks and within 0.5 Mb 5′ and 3′ of the LD blocks were found. There was only a single Refgene (Cadherin-12, *CDH12*) in the region on GGA2. There were fifteen Refgenes (*CDX1*, *CSF1R*, *NPY7R*, *FLT4*, *CANX*, *HNRNPH1*, *DGUOK*, *RUFY1*, *MAPK9*, *RASGEF1C*, *HNRNPAB*, *NME5*, *WNT8A*, *FAM13B* and *NPY6R*) located in the region on GGA13.

**Table 5:** Significant epistatic effects on MeC

Chr1	Position1	Locus1	RS#	Chr2	Position2	Locus2	RS#	Test	*P*_value
2	73,383,598	Gga_rs14204534	rs14204534	13	13,041,051	Gga_rs14998703	rs14998703	AA	3.39 × 10^−12^
2	73,383,598	Gga_rs14204534	rs14204534	13	13,361,236	Gga_rs14998801	rs14998801	AA	3.39 × 10^−12^
2	73,383,598	Gga_rs14204534	rs14204534	13	13,343,436	Gga_rs15704596	rs15704596	AA	3.39 × 10^−12^
2	73,420,901	Gga_rs14204566	rs14204566	13	13,041,051	Gga_rs14998703	rs14998703	AA	3.39 × 10^−12^
2	73,420,901	Gga_rs14204566	rs14204566	13	13,361,236	Gga_rs14998801	rs14998801	AA	3.39 × 10^−12^
2	73,420,901	Gga_rs14204566	rs14204566	13	13,343,436	Gga_rs15704596	rs15704596	AA	3.39 × 10^−12^
2	73,507,376	Gga_rs16037701	rs16037701	13	13,041,051	Gga_rs14998703	rs14998703	AA	3.44 × 10^−12^
2	73,507,376	Gga_rs16037701	rs16037701	13	13,361,236	Gga_rs14998801	rs14998801	AA	3.44 × 10^−12^
2	73,507,376	Gga_rs16037701	rs16037701	13	13,343,436	Gga_rs15704596	rs15704596	AA	3.44 × 10^−12^
2	73,559,761	GGaluGA153643	rs317095612	13	13,041,051	Gga_rs14998703	rs14998703	AA	3.44 × 10^−12^
2	73,559,761	GGaluGA153643	rs317095612	13	13,361,236	Gga_rs14998801	rs14998801	AA	3.44 × 10^−12^
2	73,559,761	GGaluGA153643	rs317095612	13	13,343,436	Gga_rs15704596	rs15704596	AA	3.44 × 10^−12^
2	73,584,220	Gga_rs14204639	rs14204639	13	13,041,051	Gga_rs14998703	rs14998703	AA	4.47 × 10^−12^
2	73,584,220	Gga_rs14204639	rs14204639	13	13,361,236	Gga_rs14998801	rs14998801	AA	4.47 × 10^−12^
2	73,584,220	Gga_rs14204639	rs14204639	13	13,343,436	Gga_rs15704596	rs15704596	AA	4.47 × 10^−12^

## Discussion

The birds from lean and fat chicken lines used in the current study had significantly different amounts of abdominal fat content and significantly different testis weight, which was an ideal population to study the genetic architecture of abdominal fat deposition and testis growth and development [19]. The birds used in the current study were 7-week-old, therefore, the results of the current study could reflect early testis development. Testis weight has been reported to be controlled by genetic factors in both chickens and mice [20–24]. Roosters with small testes often have poor fertility [25]. There have been previously reported studies on the genetics of testis development in chicken [19]. However, most of the previous studies focused on additive genetic effects, while, epistatic effects were ignored. It is unknown how important epistatic interactions are for development of the testis. In the current study, we used 475 birds from lean and fat lines to conduct epistasis analysis for testis weight and other carcass and growth traits in chickens.

The two lines used in the current study were selected for 11 years and some regions of the genome may be fixed because of selection pressure. If this occurred, it would not be possible to identify epistatic interactions in these regions of the genome. In contrast, if the two lines were crossed, it would be possible to detect epistatic effects in this intercross population. Despite this problem, some epistatic effects were detected in these two lines using epiSNP. Due to the selection applied to these population, it is possible that genomic stratification has occurred. epiSNP, which was used to conduct these analyses is capable of adjusting for family structure [18].

In this study, the SNP by SNP interaction effects for carcass and growth traits were filtered by three criteria. First, to correct for multiple testing comparisons, a Bonferroni correction was used instead of false discovering rate (FDR) method to minimize false positives. Utilization of the Bonferroni correction method will however also decrease the discovery of true interactions compared to the use of a false discovery rate correction. Therefore, the SNP X SNP interaction detected in this study may be fraction of the interactions that affect these. Second, when considering the linkage disequilibrium (LD) between the SNPs and the QTL for traits of interest, an interaction between two SNPs on the same chromosome may detect a haplotype effect but not an interaction. In other words, it is not possible to separate true interaction effects from haplotype effects if the two SNPs are located on the same chromosome. Therefore, in order to increase the power to detect the true interaction effects for the growth and carcass traits, we deleted interactions that occurred on the same chromosome [12]. Third, the smaller the number of birds in any given genotype class, the less likely it is to get a good estimate of the genotype effect. Thus, in order to increase the power to detect the true interactions, only those interactions that contained at least 10 animals in every genotype combination were considered [12]. We carried out the filter of the interactions according to these criteria in order to reduce the chances of obtaining false-positive results (type I errors).

For testis weight, a total of 421 pairs of SNP-SNP epistatic interactions were detected. These pairs of SNPs comprised 211 single SNPs, and none of these individual SNPs were identified in our previous GWAS analysis for testis weight [19]. A similar phenomenon was also detected by Wu et al. for psoriasis in human [26]. These results indicated that all SNPs on the chip should be tested for identifying potential interaction effects. In contrast, testing for interactions between SNPs that have been previously identified in GWAS is not enough.

There were 11 sub-networks with at least three nodes that were identify by using the 421 pairs of interaction effects. Sub-network1 was the biggest one and most of the interactions in Sub-network1 occurred between GGAZ and GGA21. The two regions on GGA21 and GGAZ that may harbor genes important for testis growth and development spanned 572 kb and 9.5 Mb, respectively. Three genes on GGA21 (*SDHB*, *PARK7* and *VAMP3*) and nine genes (*AGTPBP1*, *CAMK4*, *CDC14B*, *FANCC*, *FBP1*, *GNAQ*, *PTCH1*, *ROR2* and *STARD4*) on GGAZ might be important for testis growth and development based on their annotated functions. The previous result indicated that the motility and viability of sperm were positively correlated with mitochondrial *SDHB* [27]. Therefore, *SDHB* may serve as a marker of sperm quality and male fertility [28]. *PARK7* (*DJ1*) is highly expressed in human testes and has been shown to be essential for sperm maturation and fertilization [29–32]. *VAMP3* has been shown to play an important role in the process of fertilization of sperms in pig [33]. In mice, *AGTPBP1* was important for spermatogenesis, moreover, it was important for the survival of germ cells from the spermatocyte stage onward [34]. In mice and rats, the the CAMK4 gene encodes two proteins, Ca^2+^/calmodulin-dependent protein kinase IV (CaMKIV) and calspermin (CaS) [35–38]. CaMKIV is highly expressed in mouse testis and ovary and plays a essential role in male and female fertility [38–40]. *CDC14B* mutant mice were less fertile than the wild-type control [41]. Reduced fertility was reported for Fancc^−/−^ mice [42]. The expression of some proteins, including FBP1, were altered and their functions may be damaged in infertile men with unilateral varicocele [43]. The results of a previous study identified that Gnaq^d/d^ male mice were subfertile [44]. The desert hedgehog (Dhh)-null mutant male mice had less mature sperm cells and lower numbers of Leydig cells (LCs), and Dhh played an important role in spermatogenesis by acting in a paracrine manner through the Ptch1 receptor component [45–47]. In mice, female Ror2^W749FLAG/W749FLAG^ were fertile, however, Ror2^W749FLAG/W749FLAG^ male mice showed a decreased in fertility [48]. StarD6 was testis-specific expressed which indicated that it may be important for fertility [49]. StarD6 is homology to StarD4, which indicated that StarD4 may have similar function as StarD6. In Sub-network2, eight SNPs on GGA10 all interacted with the SNP (Gga_rs15834332) on GGA19, which could be seen as the hub site of Sub-network2. Thus, Gga_rs15834332 may be the important node which interacts with a 4.3 Mb region on GGA10. The Gga_rs15834332 on GGA19 was located between *CCL5* and MIR142. A total of 32 Refgenes were located in the 4.3 Mb region of GGA10. Among these genes, *TCF12* was predicted as a target gene for miR-142-5p [50]. We also detected that *TCF12* was the only gene that was predicted to be the target of miR-142-5p using three packages online. Therefore, it is proposed that miR-142-5p and *TCF12* might work together to regulate the reproductive function of male broilers. Furthermore, *TCF12* was a partner of *TCF21*, which was detected as an important gene for testis growth and development in our previous GWAS result [19].

For MeC, 15 pairs of SNPs with significant epistatic effects were detected, which indicated an interaction between GGA2 and GGA13. It is proposed that *CDH12* on GGA2 and *WNT8A* on GGA13, which are both located in the same cadherin signaling pathway, may be important for bone growth. It had been shown that the pathway was involved in many biological processes, such as development, neurogenesis, cell adhesion, and inflammation, and also involved in many disease, such as cancer [51].

## Conclusions

In the current study, a large number of epistatic interactions were found to be significantly associated with testis weight in chicken. It appears that miR-142-5p along with its target gene *TCF12*, and some other genes in GGA21 and GGAZ (*SDHB*, *PARK7*, *VAMP3*, *AGTPBP1*, *CAMK4*, *CDC14B*, *FANCC*, *FBP1*, *GNAQ*, *PTCH1*, *ROR2* and *STARD4*) might be important for testis growth and development. In contrast, very few significant epistatic interactions were identified for other carcass and growth traits. These results indicate that epistatic interaction may play very different roles in the control of phenotypic variation for different traits in chickens.

## Additional files

Additional file 1: Figure S1. Phenotypic distribution of the carcass and growth traits in the lean and fat lines, respectively. The red and light blue bars represent the traits in the lean and fat lines, respectively. The dark blue bars represent the overlap of the traits between the two lines. (PDF 322 kb)

Additional file 2: Table S1. The 421 pairs of SNPs with significant interaction effects on testis weight (TeW). (XLSX 67 kb)

Additional file 3: Figure S2. The phenotypic distributions of the four different genotype classes for the most significant AA effect between GGaluGA22768 on GGA3 and Gga_rs14722408 on GGA10. (PDF 122 kb)

Additional file 4: Figure S3. Epistatic network among SNPs that affect testis weight (TeW). Each node represents a SNP. The chromosome in which a given SNP is located is shown within the circle. A pair of SNPs connected by an edge had a significant interaction. The colors of the nodes represent the *P*-value of an interaction (*P* < 1.0 × 10^−16^ = red; *P* < 1.0 × 10^−15^ = blue; *P* < 1.0 × 10^−14^ = green; *P* < 1.0 × 10^−13^ = white). The color of the edge indicates the type of epistatic effect (AA = red; AD = purple; DA = blue; DD = green). (PDF 1222 kb)

Additional file 5: Figure S4. The LD blocks of the SNPs on every chromosome in Sub-network1 for testis weight (TeW). This information was used to simply Sub-network1. (PDF 619 kb)

## Abbreviations

GO: Gene ontology
GWAS: Genome–wide association study
KEGG: Kyoto encyclopedia of genes and genomes
LD: Linkage disequilibrium
QTL: Quantitative trait loci
SNP: Single nucleotide polymorphism
