# Supplementary material for: Identification of genome-wide SNP-SNP interactions associated with important traits in chicken
Source: BMC Genomics. 2017 Nov 21;18:892. doi: 10.1186/s12864-017-4252-y (PMC5698929; doi:10.1186/s12864-017-4252-y)
Supplement: Supplementary file 1 — Phenotypic distribution of the carcass and growth traits in the lean and fat lines, respectively. The red and light blue bars represent the traits in the lean and fat lines, respectively. The dark blue bars represent the overlap of the traits between the two lines. (PDF 322 kb) [file 12864_2017_4252_MOESM1_ESM.pdf]

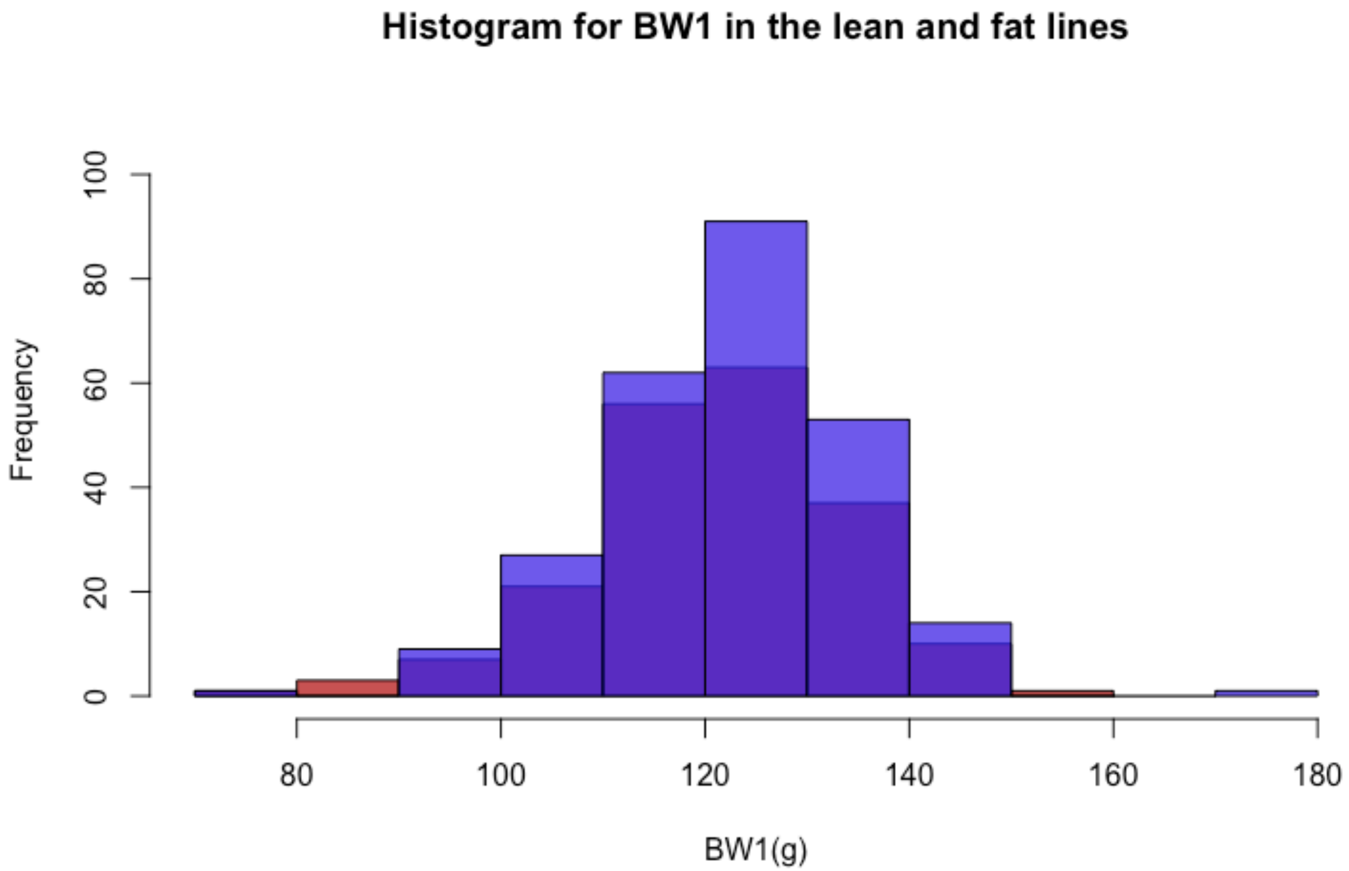
Histogram for BW1 in the lean and fat lines
Frequency
0
20
40
60
80
100
80
100
120
140
160
180
BW1(g)

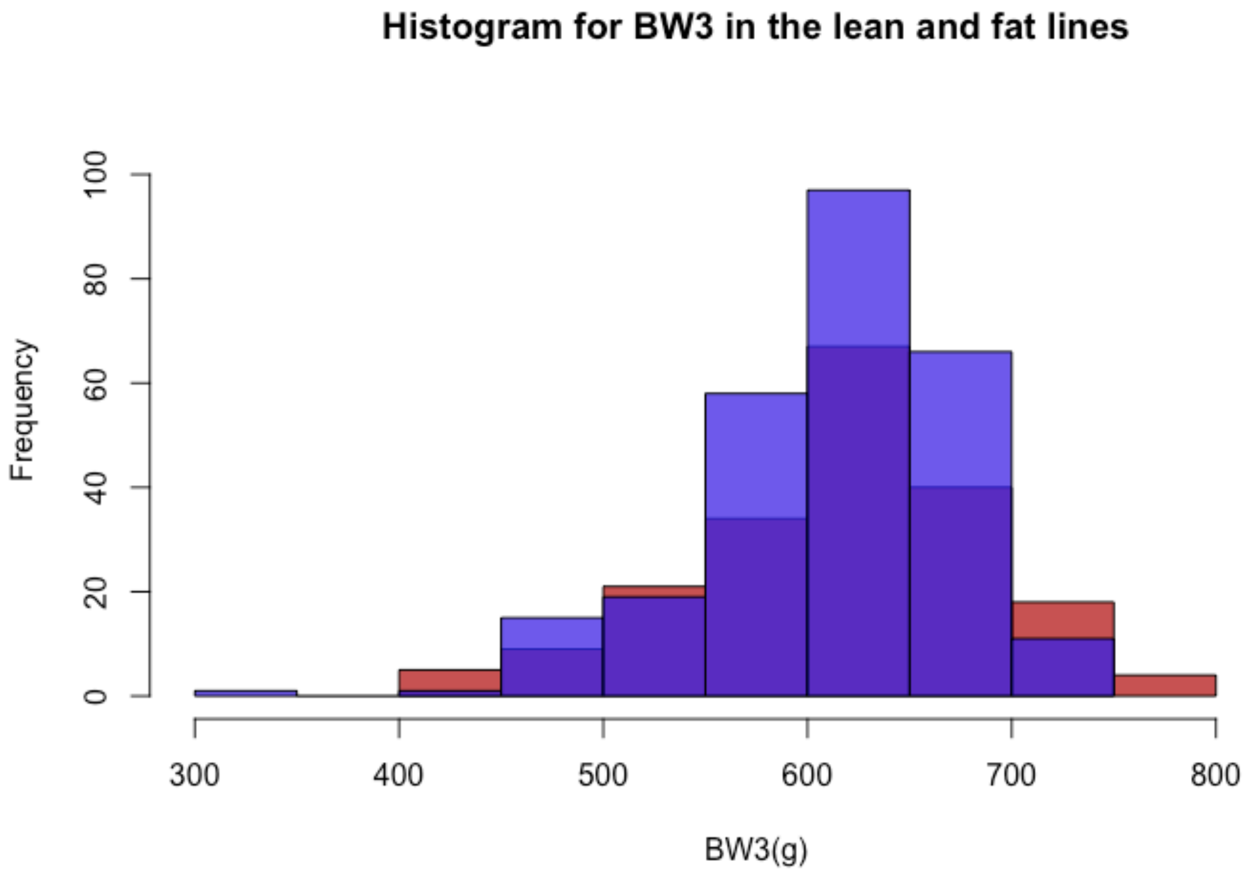
Histogram for BW3 in the lean and fat lines
Frequency
0
20
40
60
80
100
300
400
500
600
700
800
BW3(g)

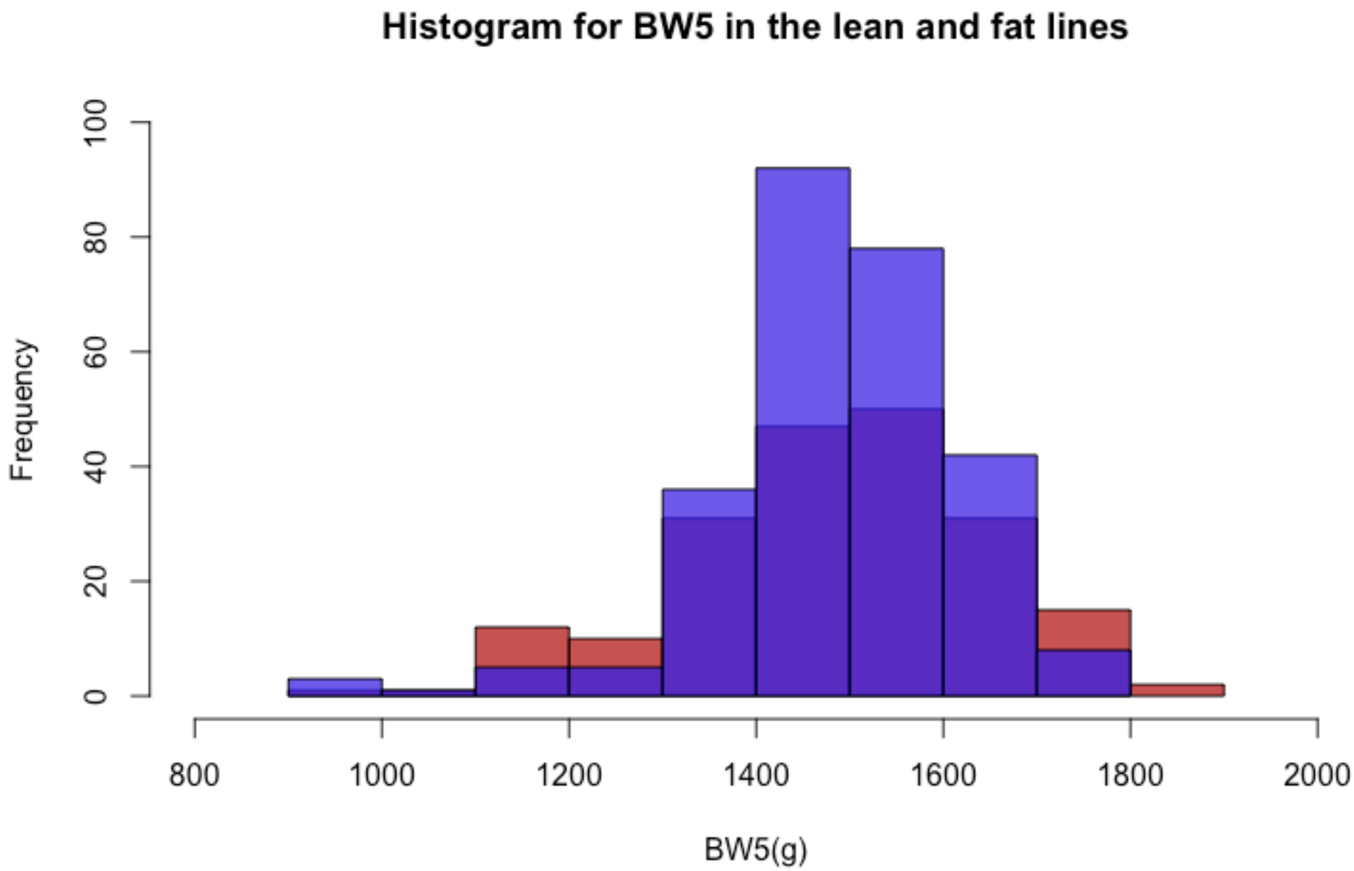
Histogram for BW5 in the lean and fat lines
Frequency
0
20
40
60
80
100
800
1000
1200
1400
1600
1800
2000
BW5(g)

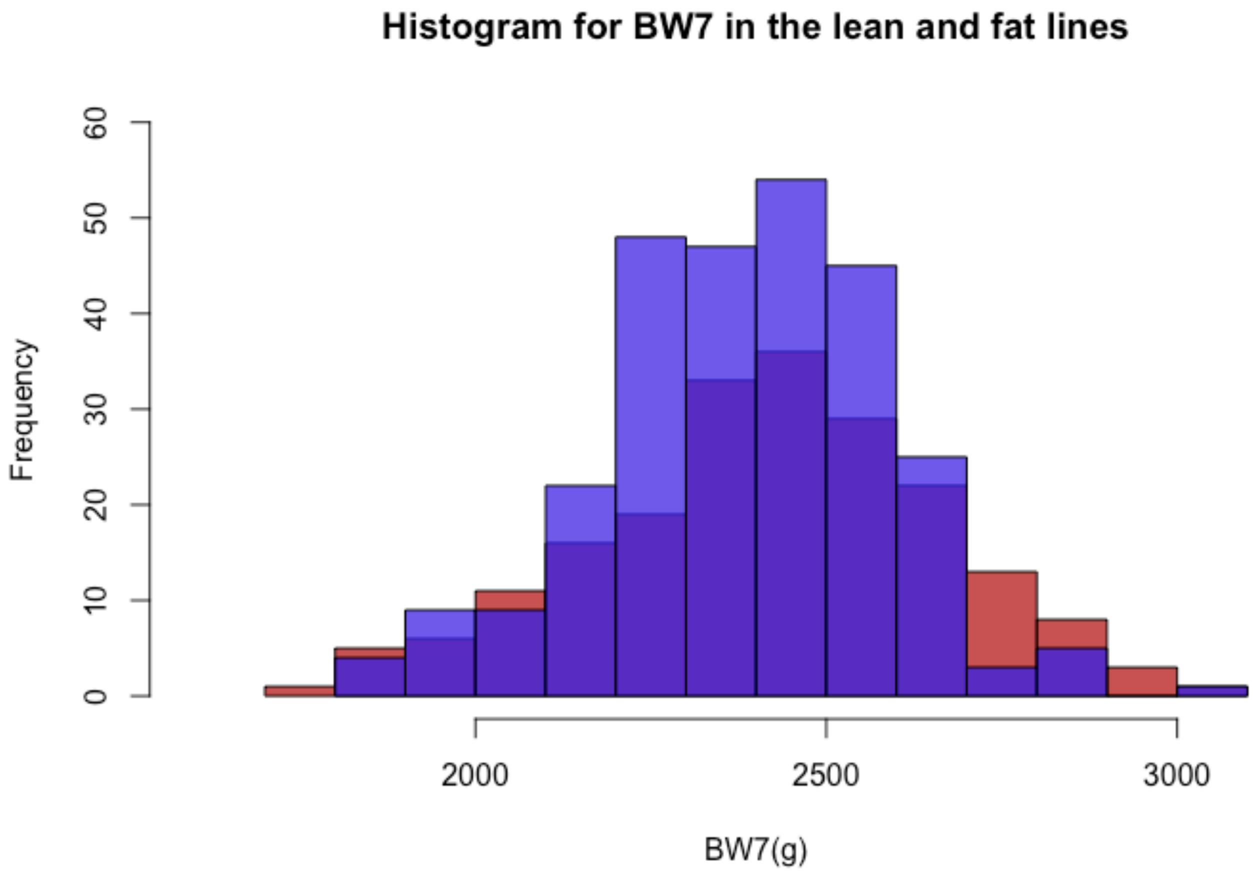
Histogram for BW7 in the lean and fat lines
Frequency
0
10
20
30
40
50
60
2000
2500
3000
BW7(g)

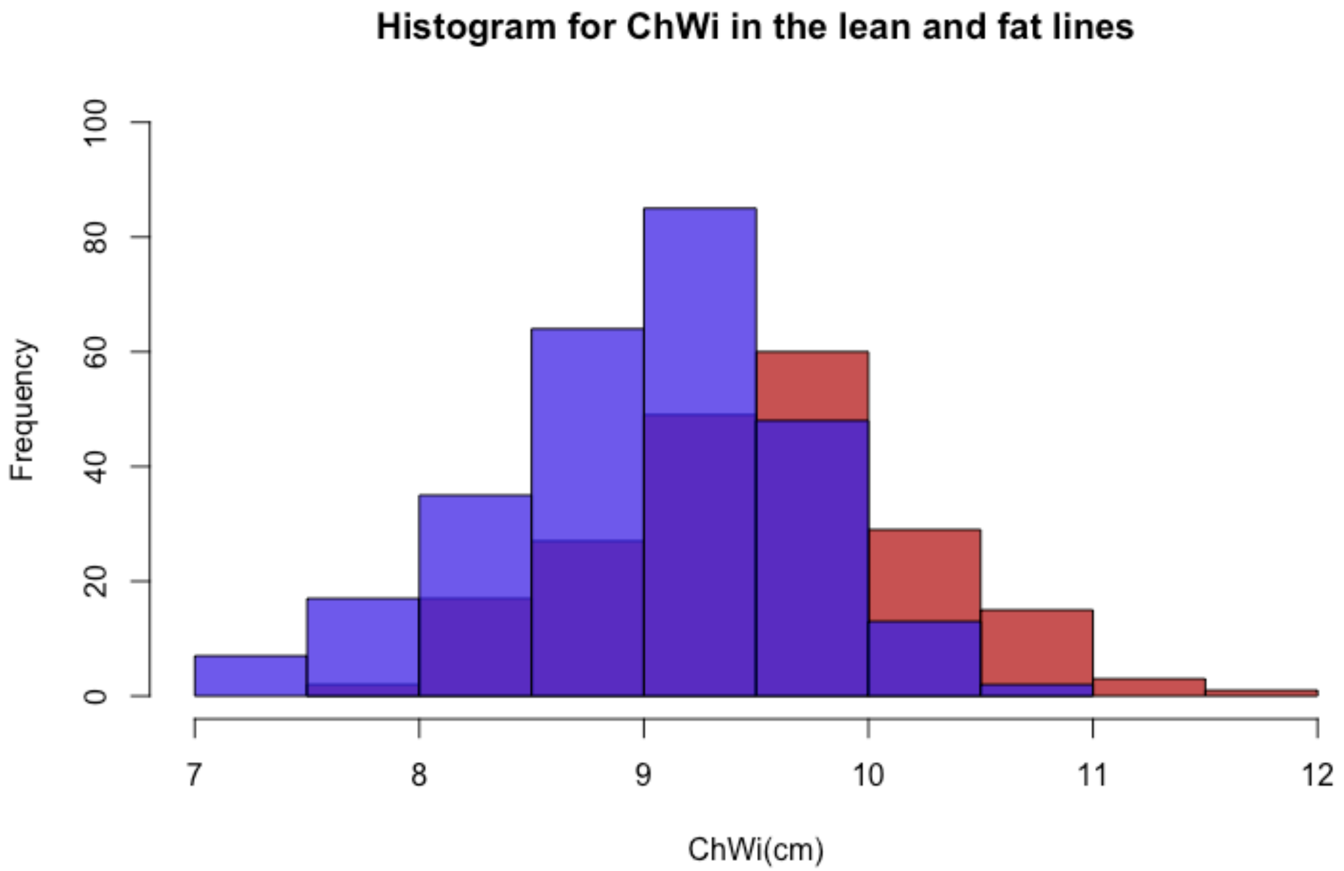
Histogram for ChWi in the lean and fat lines
Frequency
0
20
40
60
80
100
7
8
9
10
11
12
ChWi(cm)

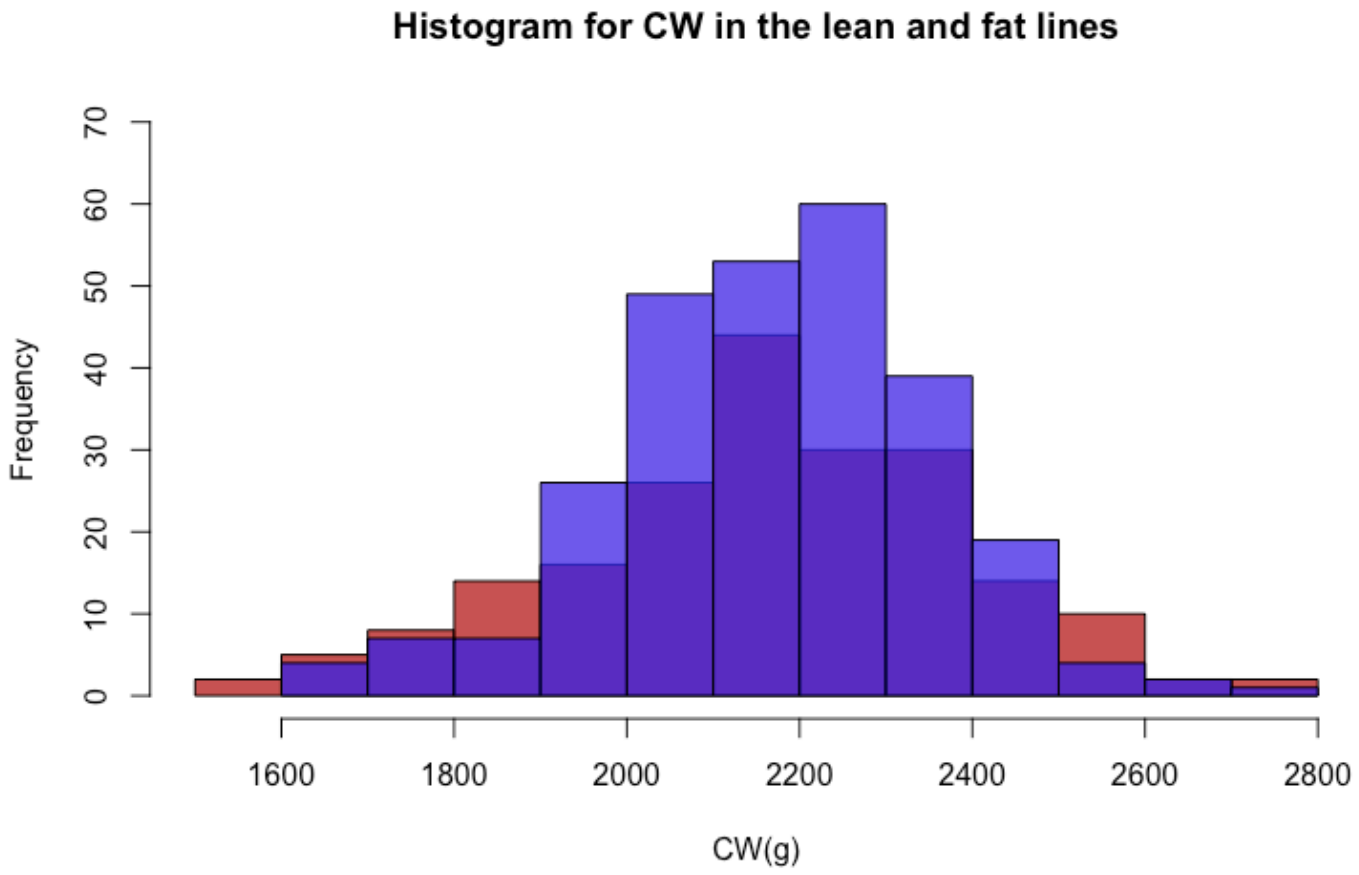
Histogram for CW in the lean and fat lines
Frequency
0
10
20
30
40
50
60
70
1600
1800
2000
2200
2400
2600
2800
CW(g)

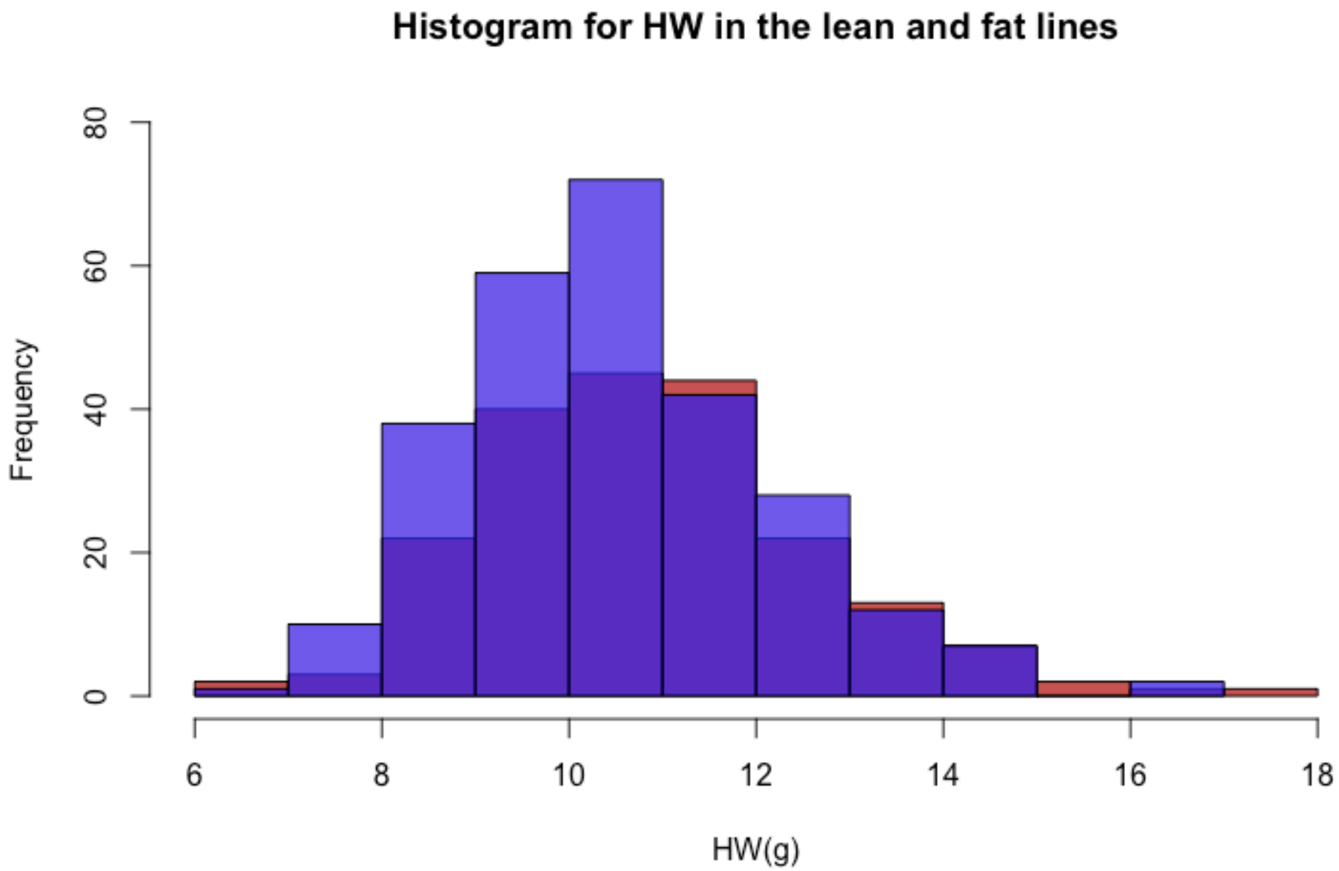
Histogram for HW in the lean and fat lines
Frequency
0
20
40
60
80
6
8
10
12
14
16
18
HW(g)

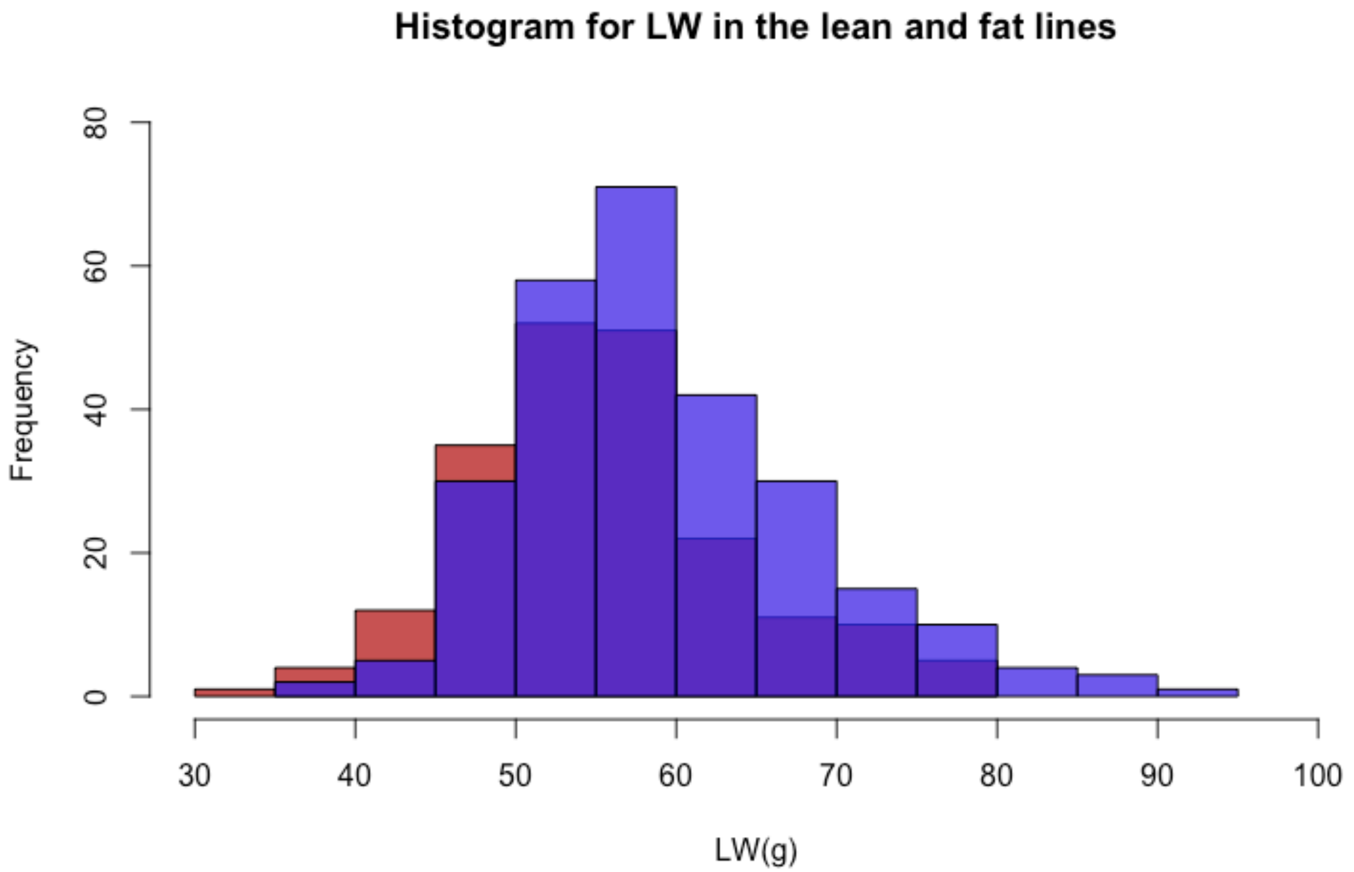
Histogram for LW in the lean and fat lines
Frequency
0
20
40
60
80
30
40
50
60
70
80
90
100
LW(g)

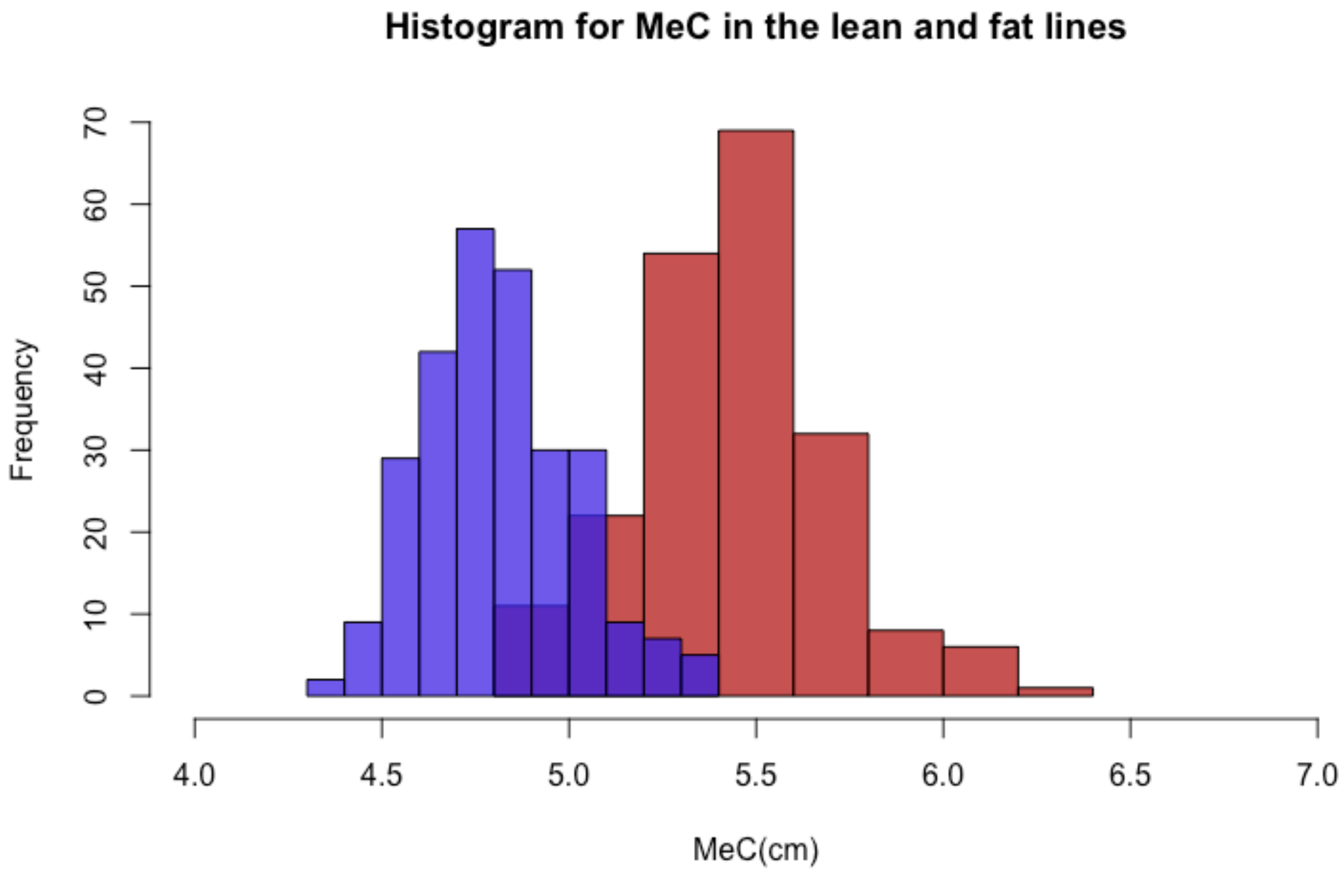

Histogram for MeC in the lean and fat lines
Frequency
0
10
20
30
40
50
60
70
4.0
4.5
5.0
5.5
6.0
6.5
7.0
MeC(cm)

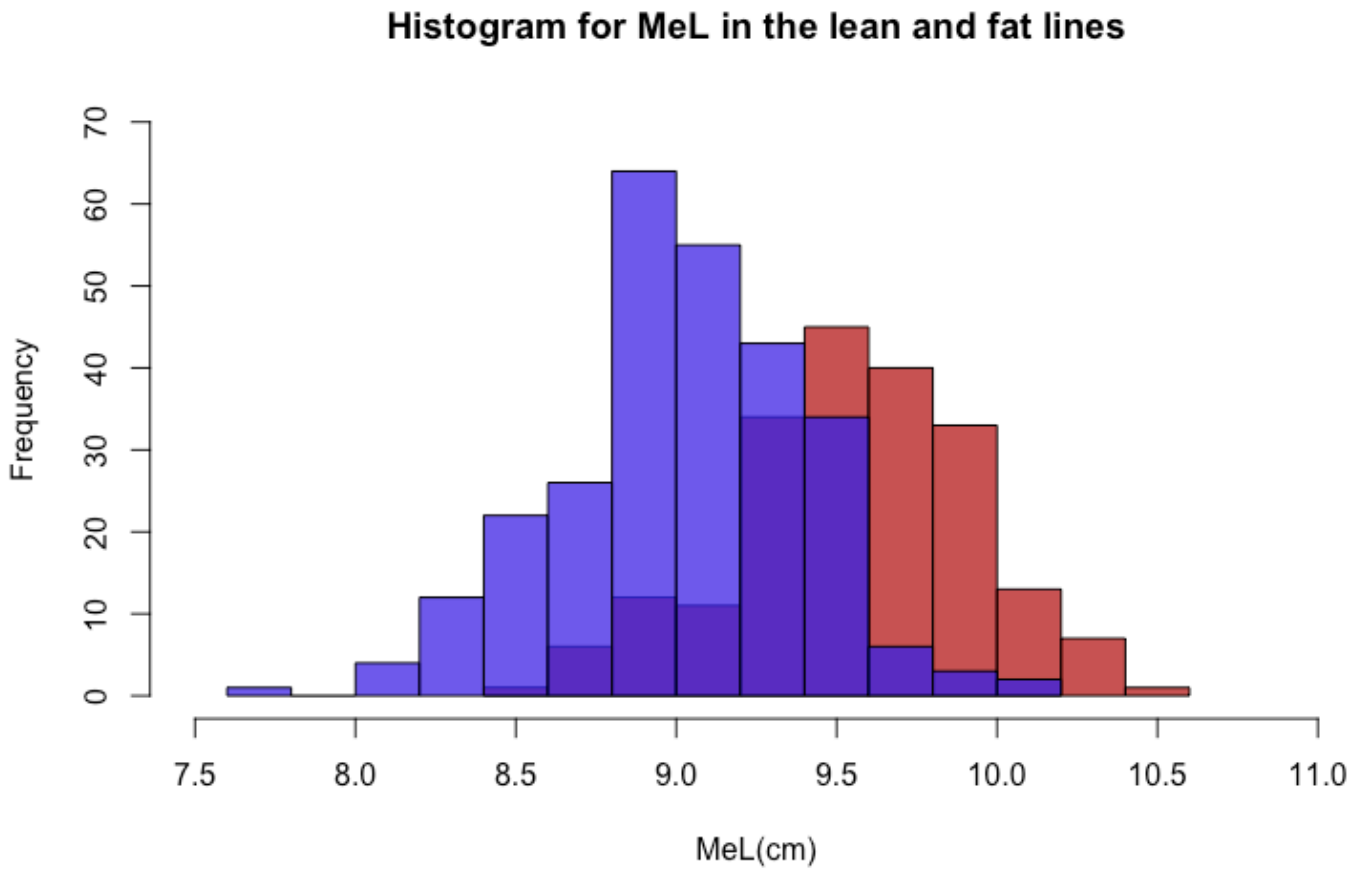
Histogram for MeL in the lean and fat lines
Frequency
0
10
20
30
40
50
60
70
7.5
8.0
8.5
9.0
9.5
10.0
10.5
11.0
MeL(cm)

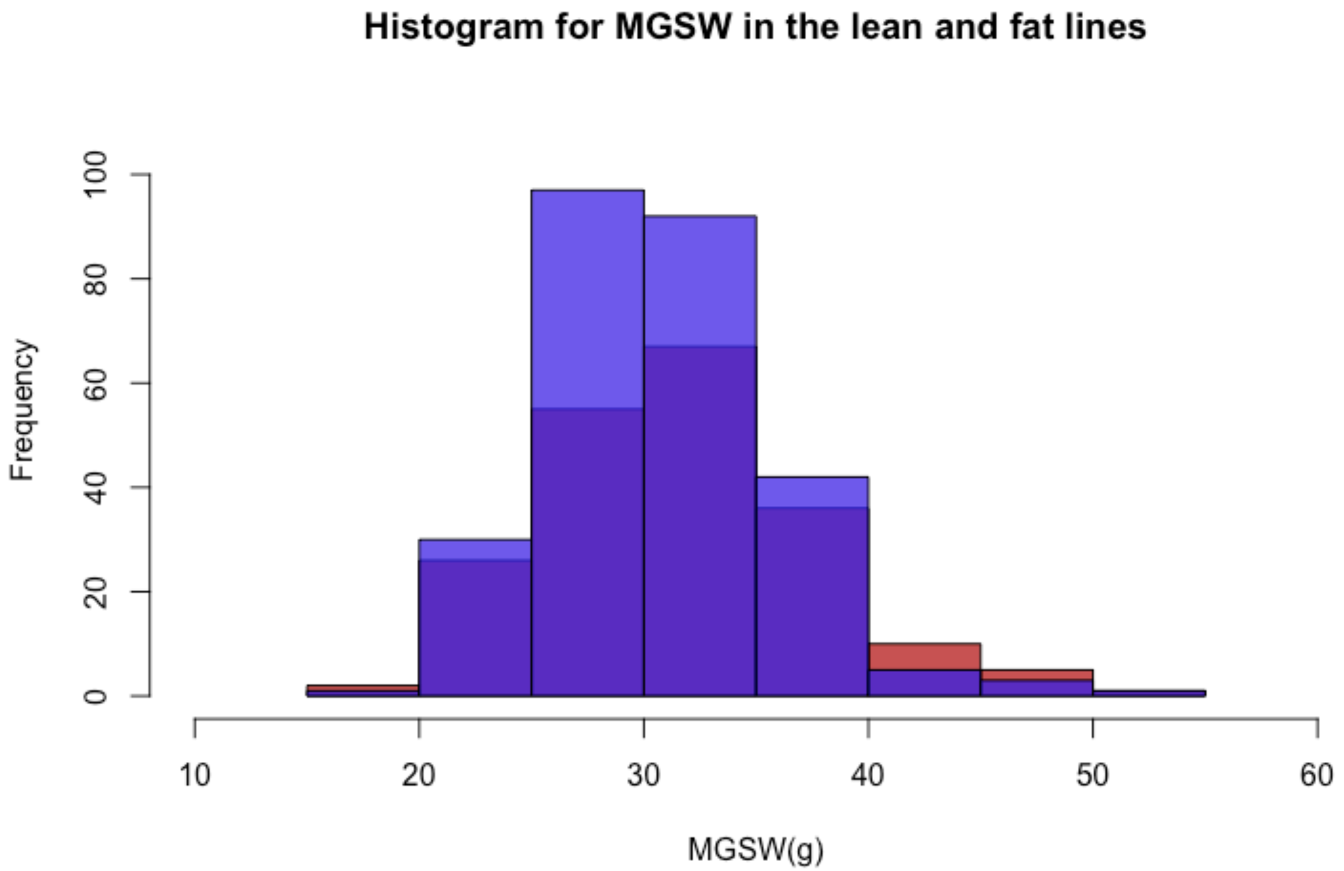
Histogram for MGSW in the lean and fat lines
Frequency
0
20
40
60
80
100
10
20
30
40
50
60
MGSW(g)

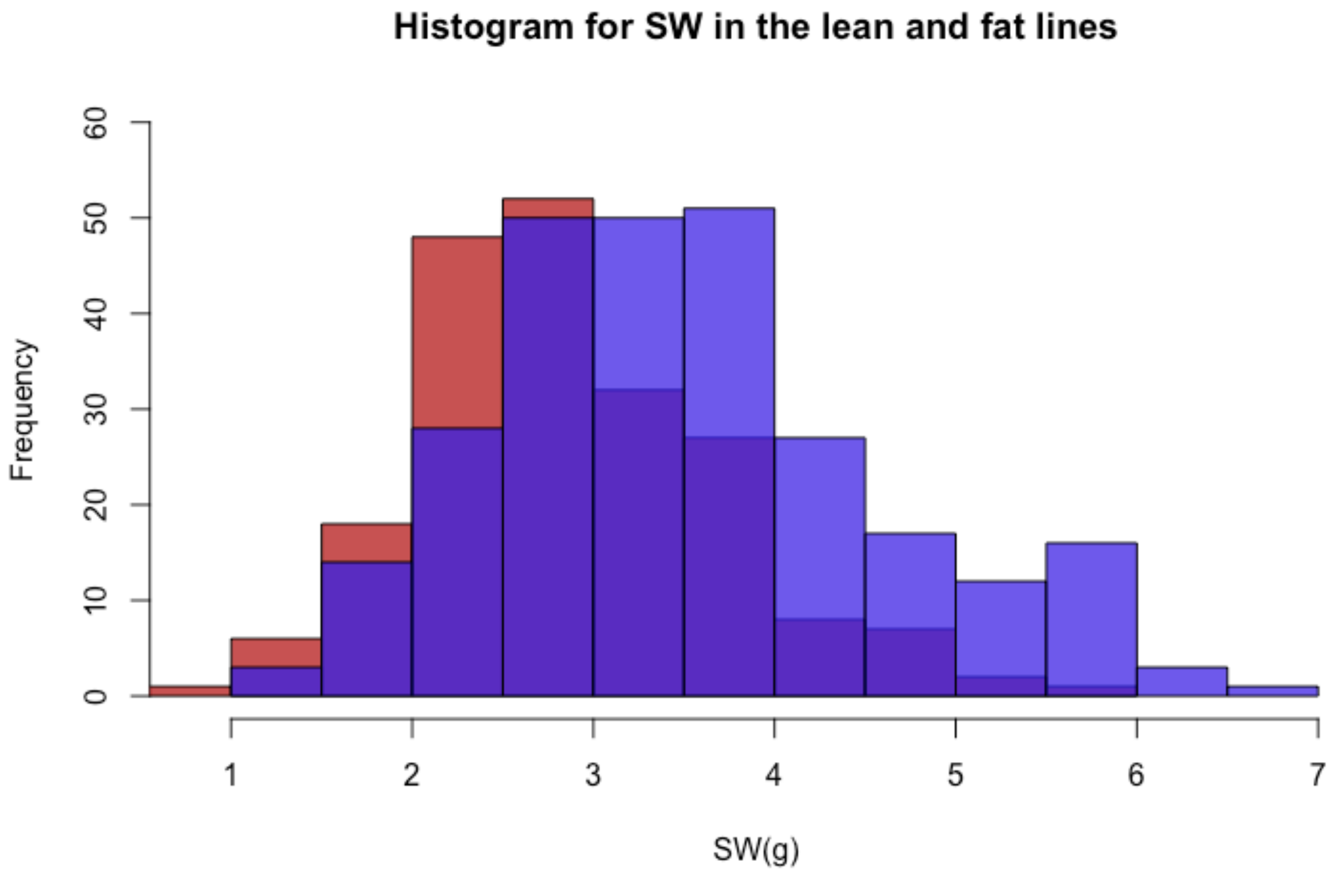
Histogram for SW in the lean and fat lines
Frequency
0
10
20
30
40
50
60
1
2
3
4
5
6
7
SW(g)

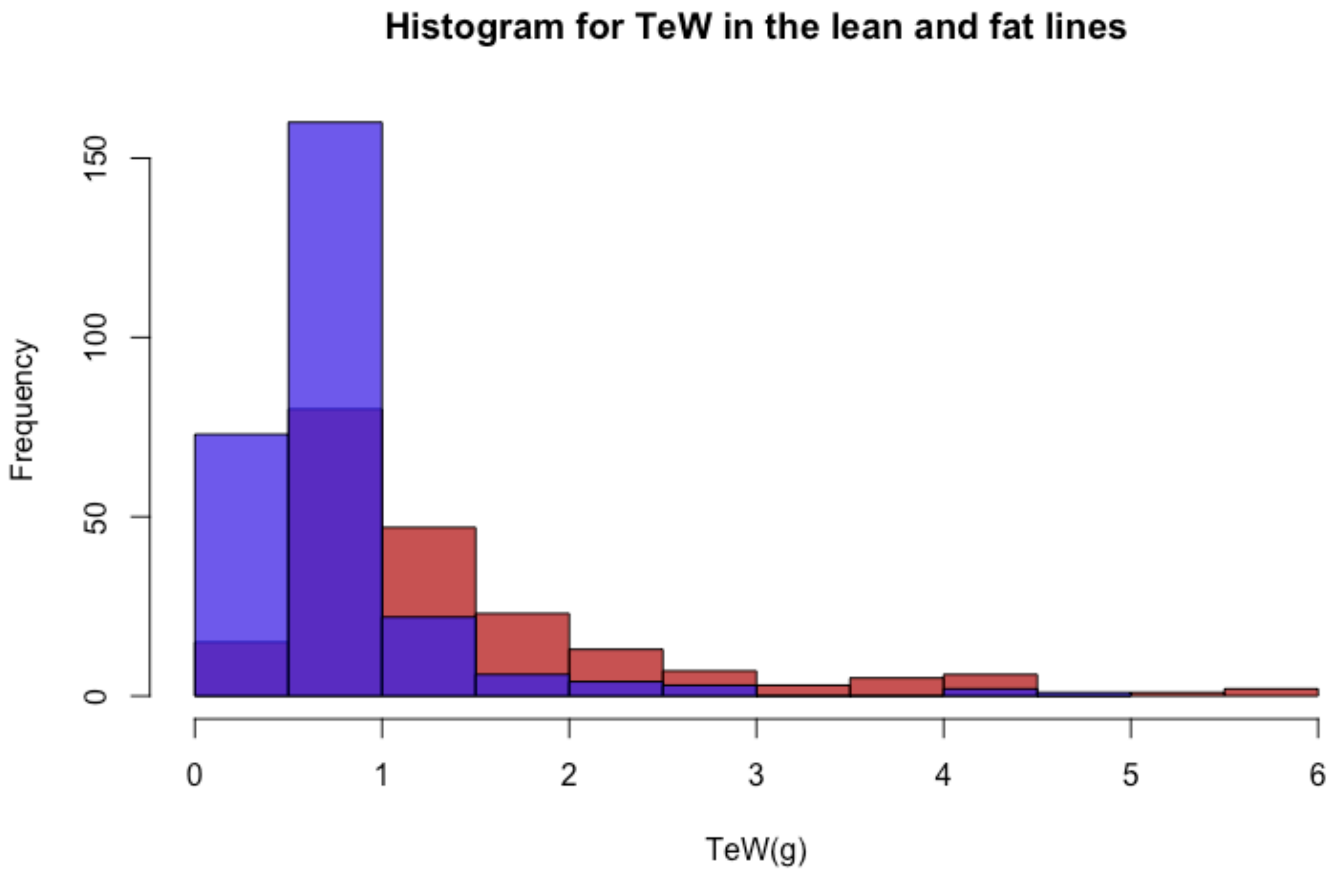

Histogram for TeW in the lean and fat lines
Frequency
0
50
100
150
0
1
2
3
4
5
6
TeW(g)
