# Supplementary material for: Identification of genome-wide SNP-SNP interactions associated with important traits in chicken
Source: BMC Genomics. 2017 Nov 21;18:892. doi: 10.1186/s12864-017-4252-y (PMC5698929; doi:10.1186/s12864-017-4252-y)
Supplement: Supplementary file 3 — The phenotypic distributions of the four different genotype classes for the most significant AA effect between GGaluGA22768 on GGA3 and Gga_rs14722408 on GGA10. (PDF 122 kb) [file 12864_2017_4252_MOESM3_ESM.pdf]

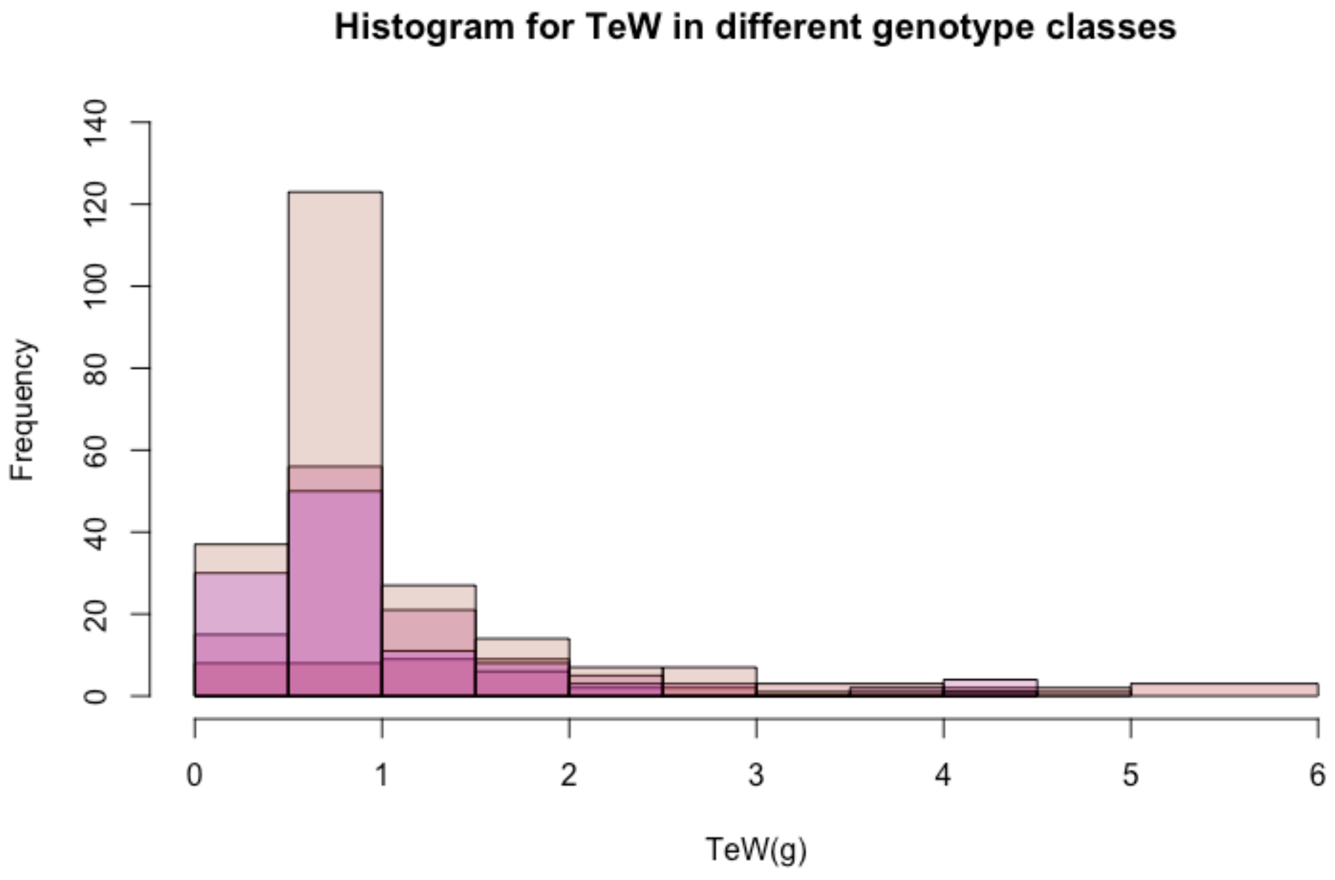

Histogram for TeW in different genotype classes
Frequency
0
20
40
60
80
100
120
140
0
1
2
3
4
5
6
TeW(g)

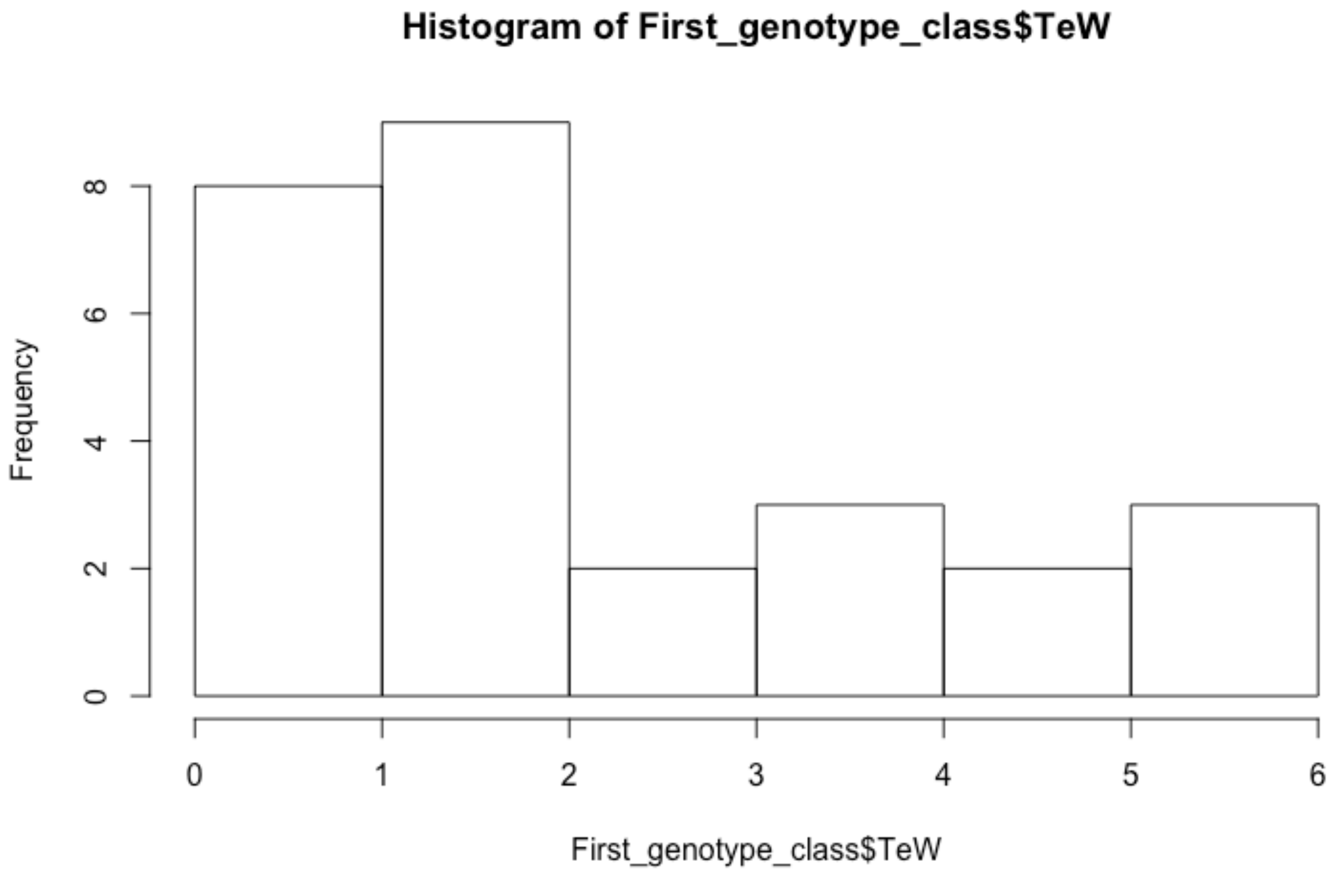

Histogram of First_genotype_class$TeW
Frequency
0
2
4
6
8
0
1
2
3
4
5
6
First_genotype_class$TeW

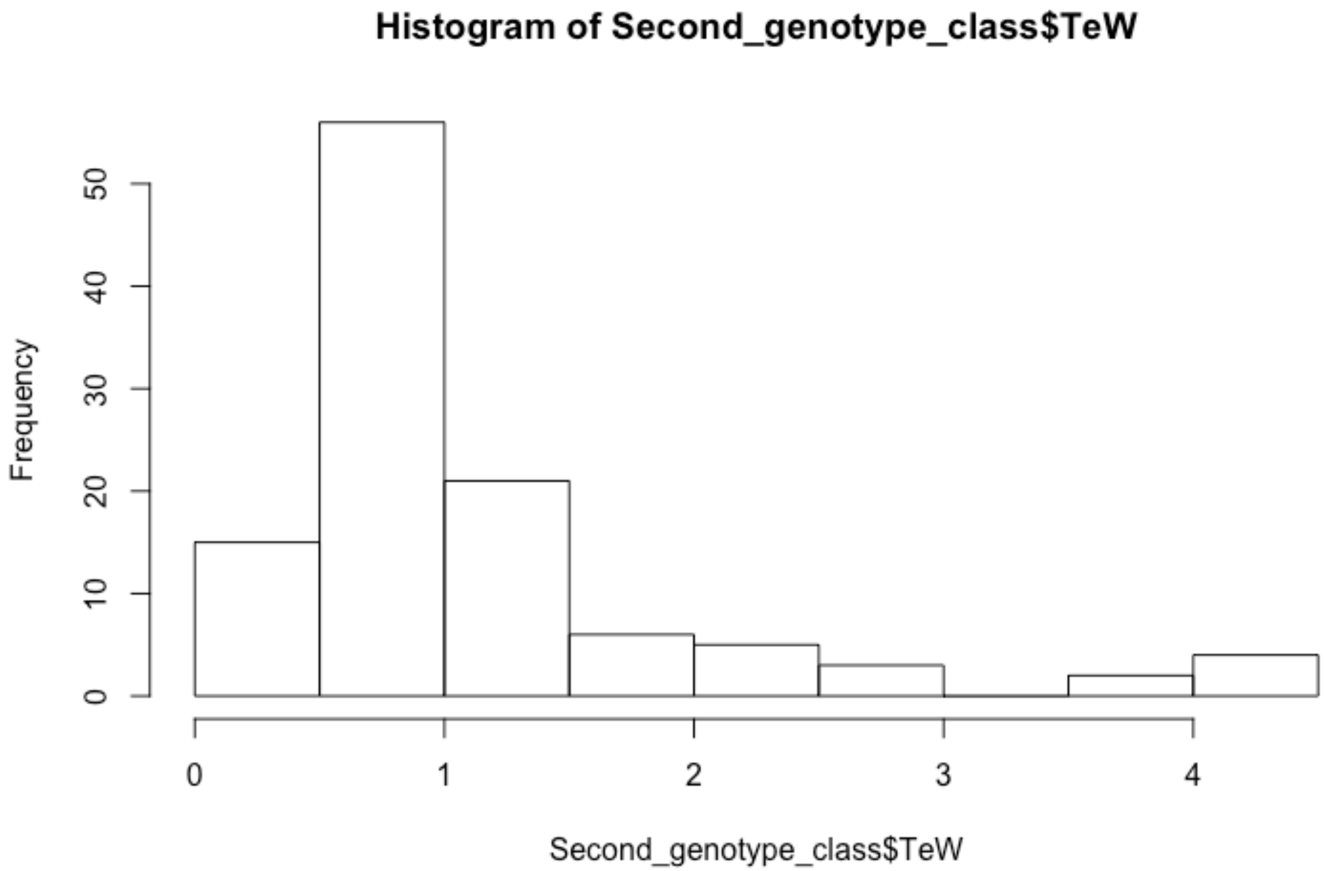
Histogram of Second_genotype_class$TeW
Frequency
0
10
20
30
40
50
0
1
2
3
4
Second_genotype_class$TeW

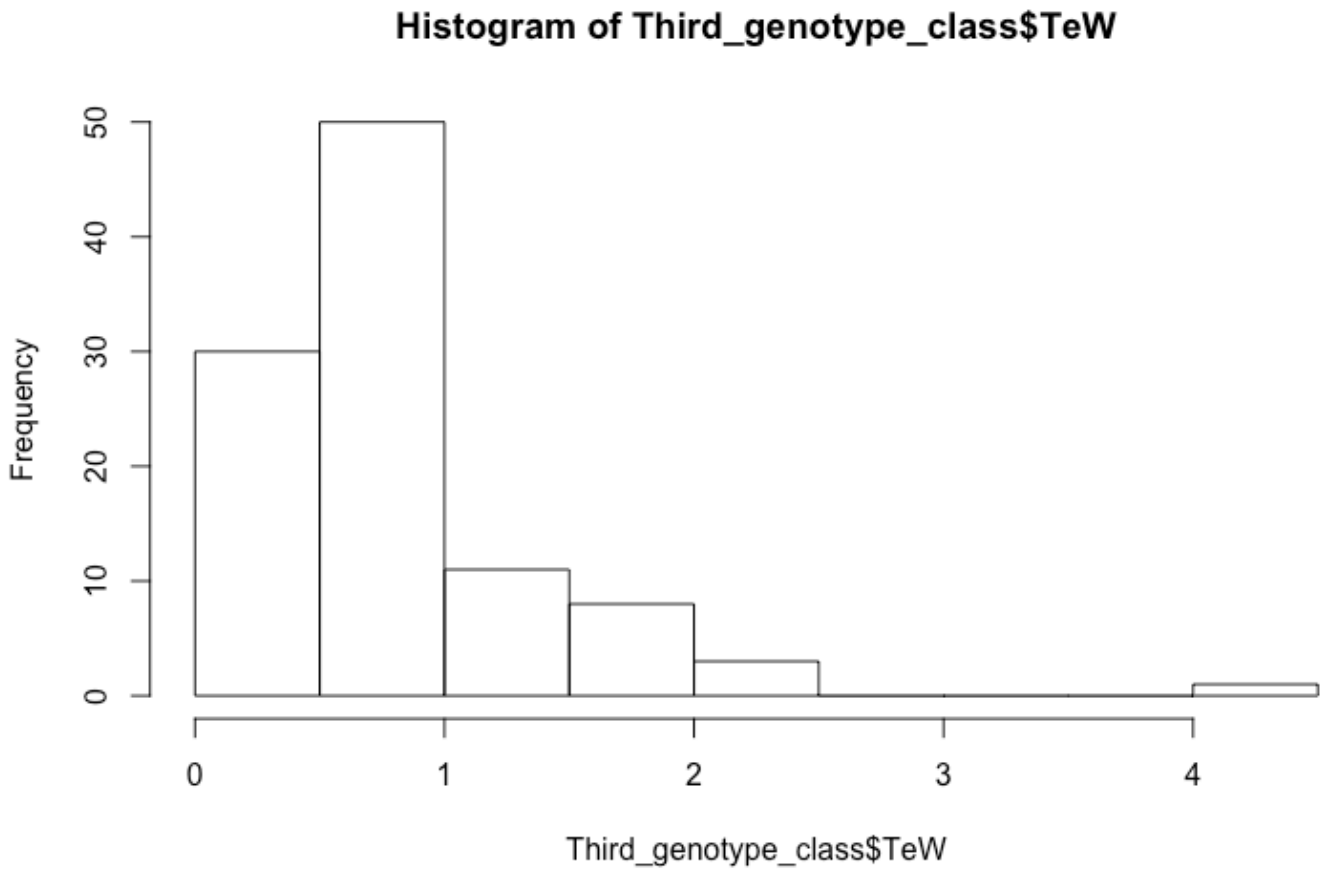

Histogram of Third_genotype_class$TeW
Frequency
0
10
20
30
40
50
0
1
2
3
4
Third_genotype_class$TeW

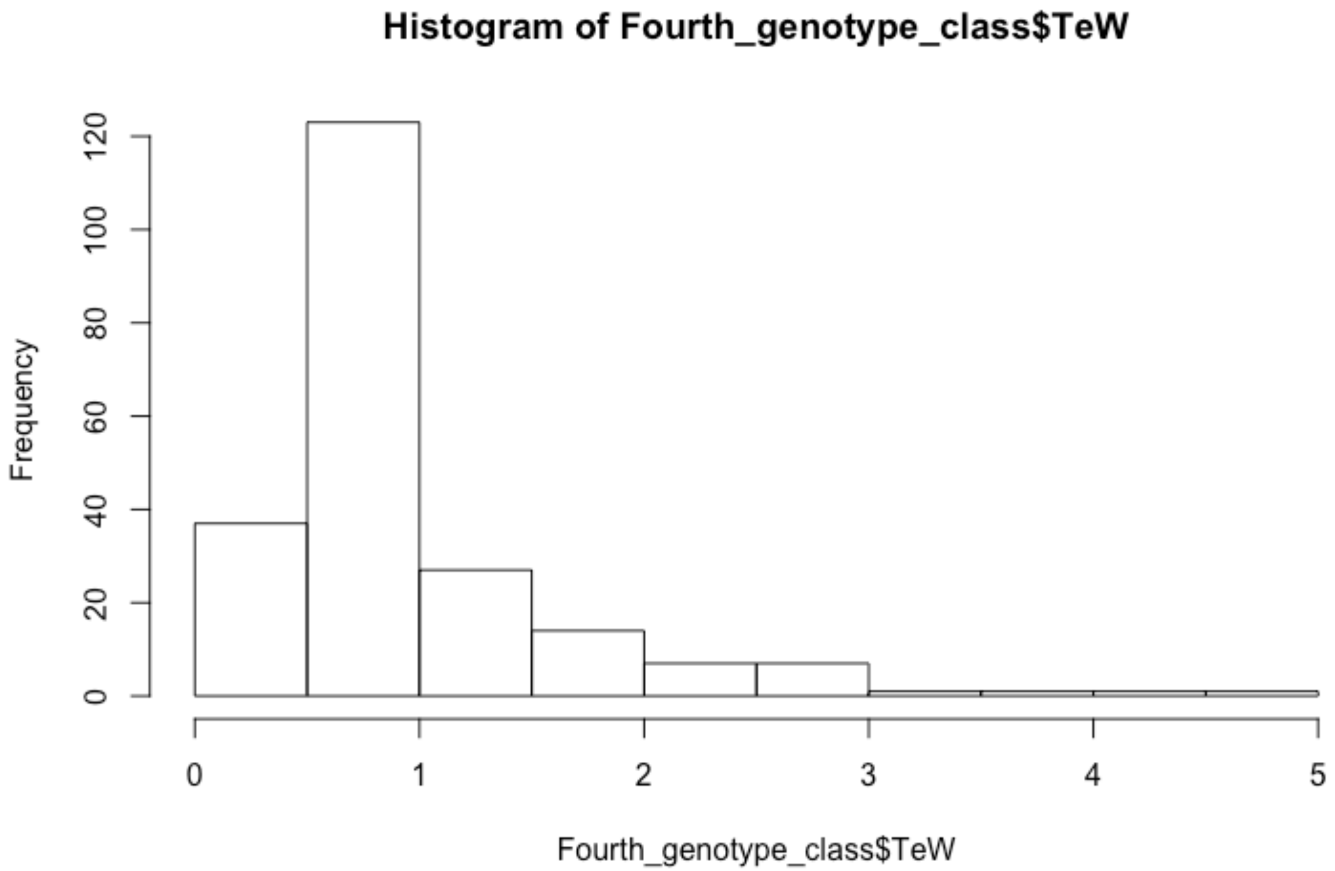
Histogram of Fourth_genotype_class$TeW
Frequency
0
20
40
60
80
100
120
0
1
2
3
4
5
Fourth_genotype_class$TeW
