# Supplementary material for: Identification of genome-wide SNP-SNP interactions associated with important traits in chicken
Source: BMC Genomics. 2017 Nov 21;18:892. doi: 10.1186/s12864-017-4252-y (PMC5698929; doi:10.1186/s12864-017-4252-y)
Supplement: Supplementary file 4 — Epistatic network among SNPs that affect testis weight (TeW). Each node represents a SNP. The chromosome in which a given SNP is located is shown within the circle. A pair of SNPs connected by an edge had a significant interaction. The colors of the nodes represent the P-value of an interaction (P < 1.0 × 10−16 = red; P < 1.0 × 10−15 = blue; P < 1.0 × 10−14 = green; P < 1.0 × 10−13 = white). The color of the edge indicates the type of epistatic effect (AA = red; AD = purple; DA = blue; DD = green). (PDF 1222 kb) [file 12864_2017_4252_MOESM4_ESM.pdf]

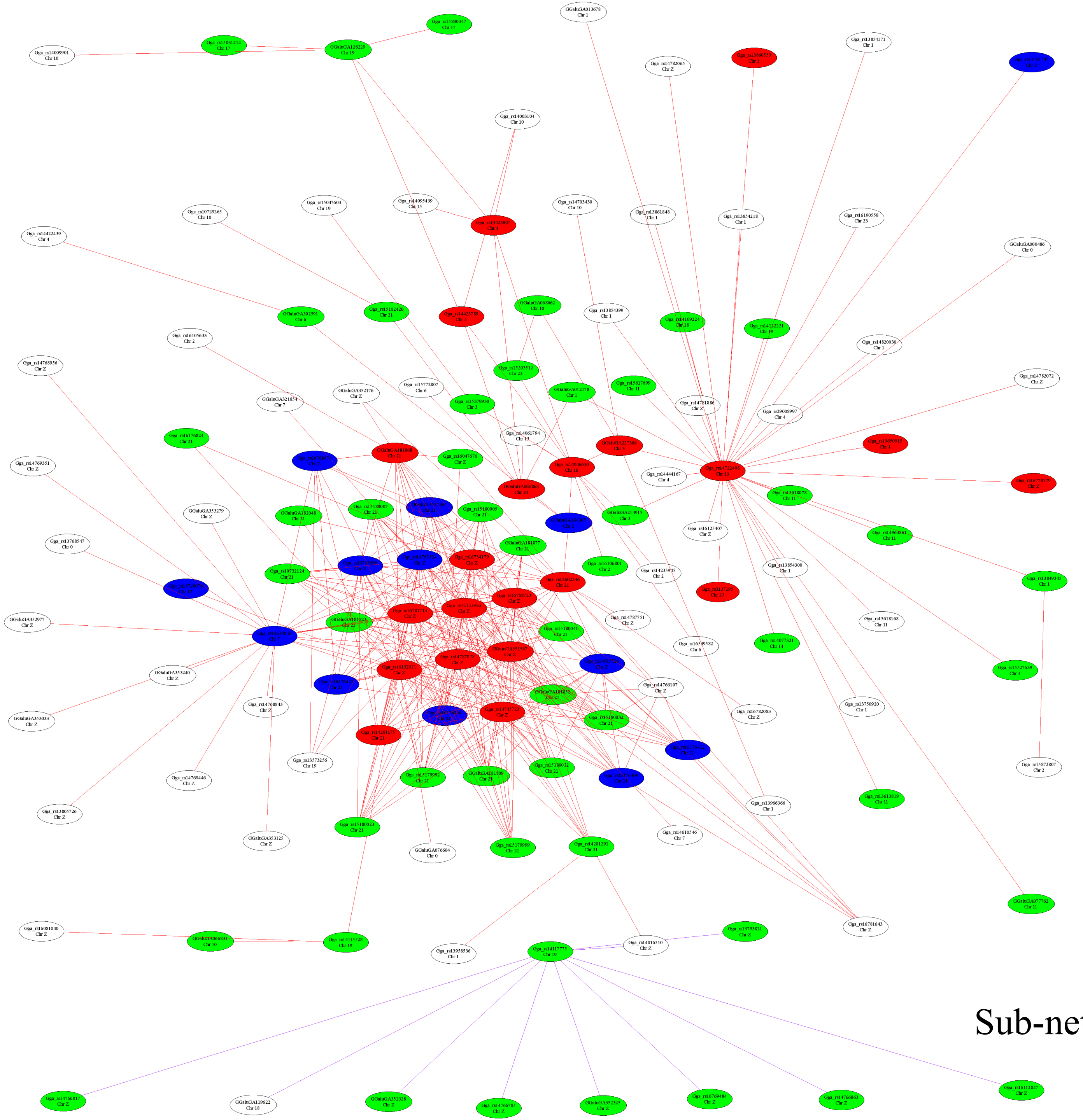

Sub-network1

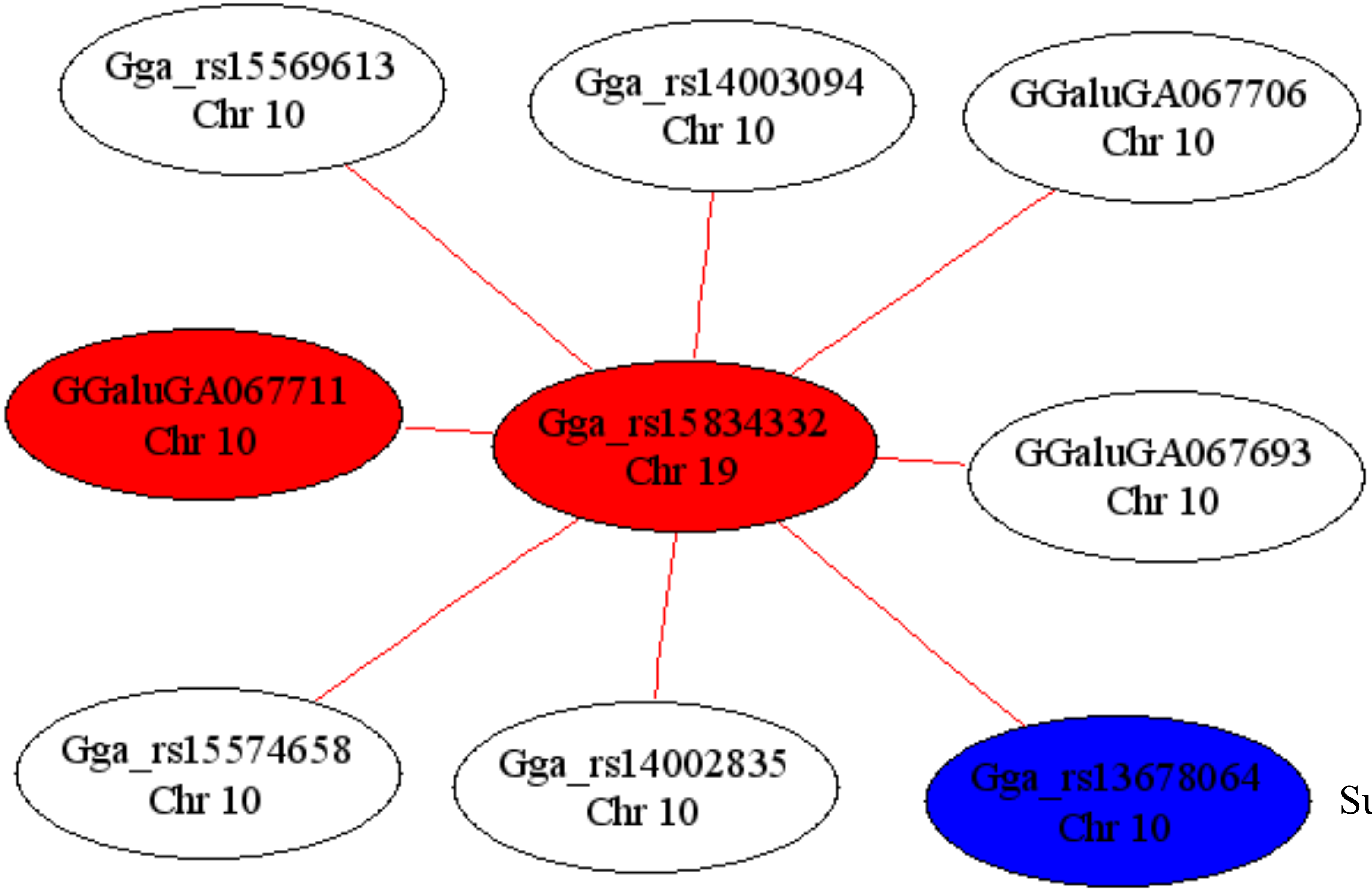

Sub-network2

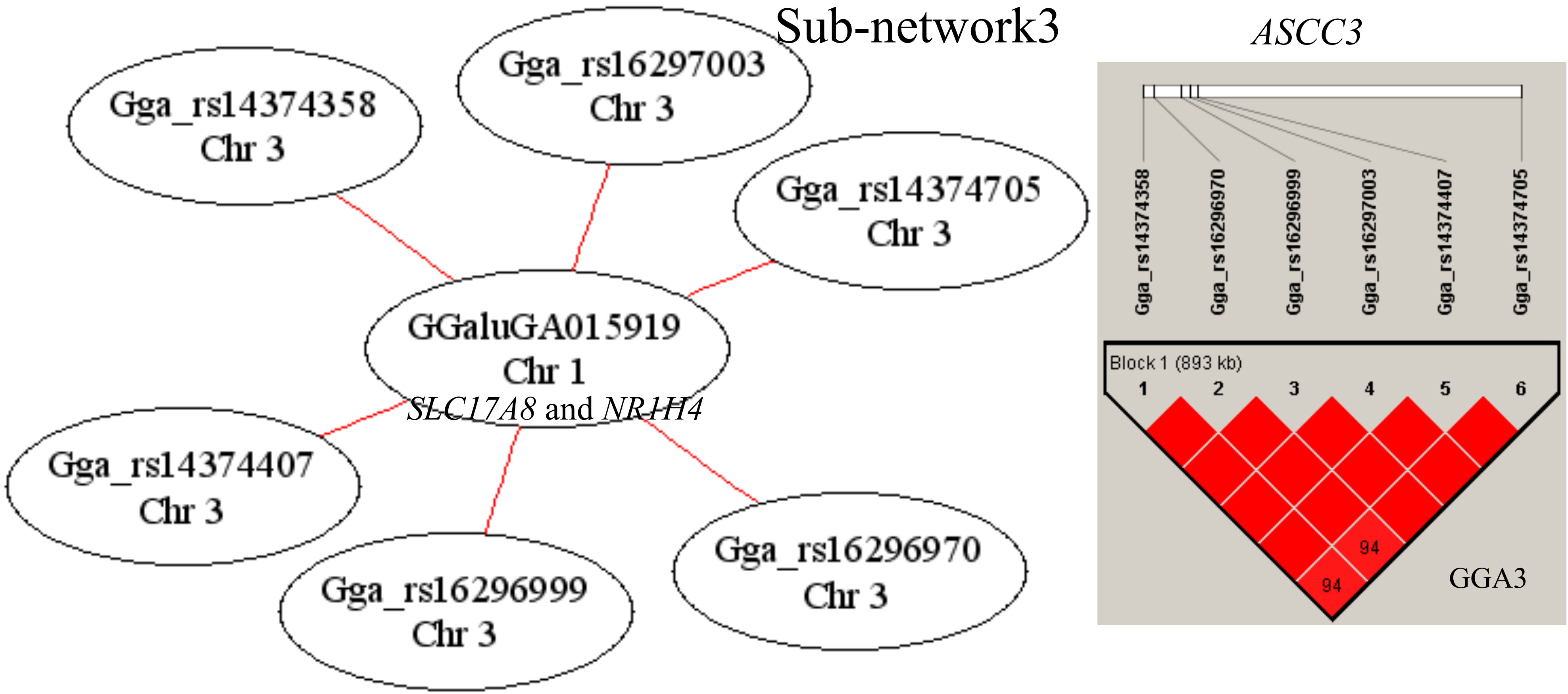
Sub-network3
Gga_rs14374358
Chr 3
Gga_rs16297003
Chr 3
Gga_rs14374705
Chr 3
GGaluGA015919
Chr 1
SLC17A8 and NR1H4
Gga_rs14374407
Chr 3
Gga_rs16296999
Chr 3
Gga_rs16296970
Chr 3
ASCC3
Gga_rs14374358
Gga_rs16296970
Gga_rs16296999
Gga_rs16297003
Gga_rs14374407
Gga_rs14374705
Block 1 (893 kb)
1
2
3
4
5
6
94
94
GGA3

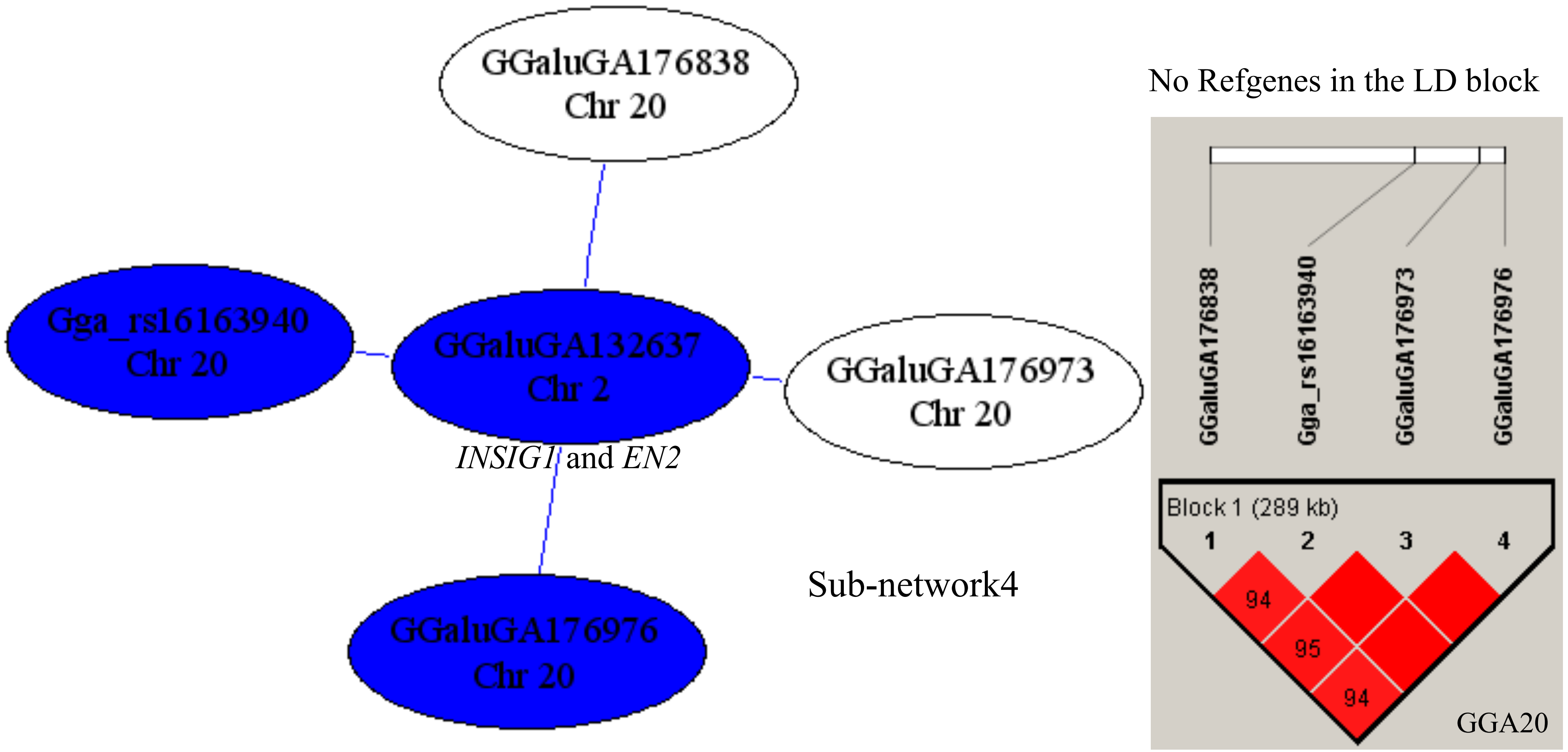
GGaluGA176838
Chr 20
Gga_rs16163940
Chr 20
GGaluGA132637
Chr 2
INSIG1 and EN2
GGaluGA176973
Chr 20
GGaluGA176976
Chr 20
Sub-network4
No Refgenes in the LD block
GGaluGA176838
Gga_rs16163940
GGaluGA176973
GGaluGA176976
Block 1 (289 kb)
1
2
3
4
94
95
94
GGA20

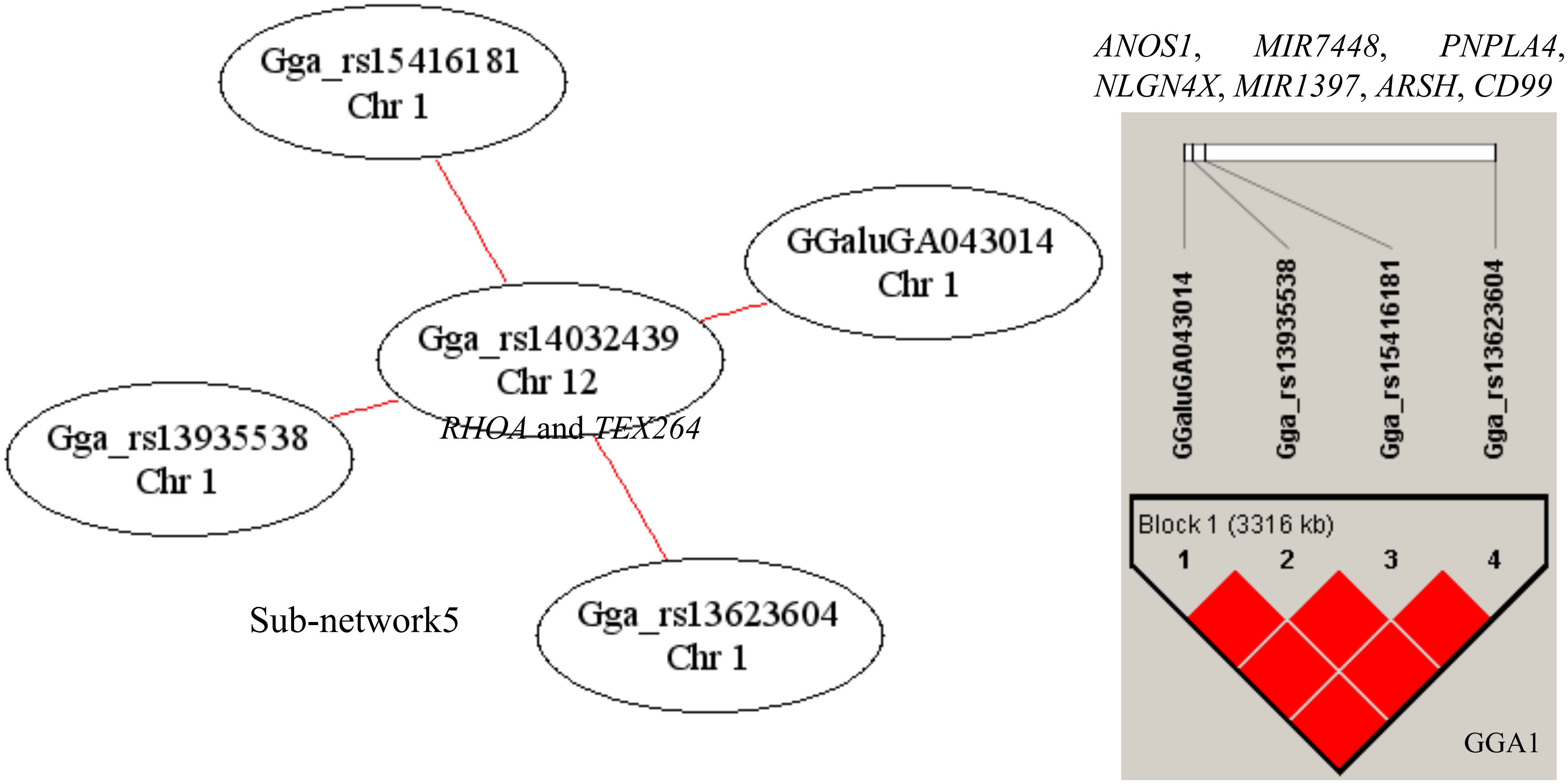
Gga_rs15416181
Chr 1
GGaluGA043014
Chr 1
Gga_rs14032439
Chr 12
RHOA and TEX264
Gga_rs13935538
Chr 1
Gga_rs13623604
Chr 1
Sub-network5
ANOS1, MIR7448, PNPLA4,
NLGN4X, MIR1397, ARSH, CD99
GGaluGA043014
Gga_rs13935538
Gga_rs15416181
Gga_rs13623604
Block 1 (3316 kb)
1
2
3
4
GGA1

Sub-network6

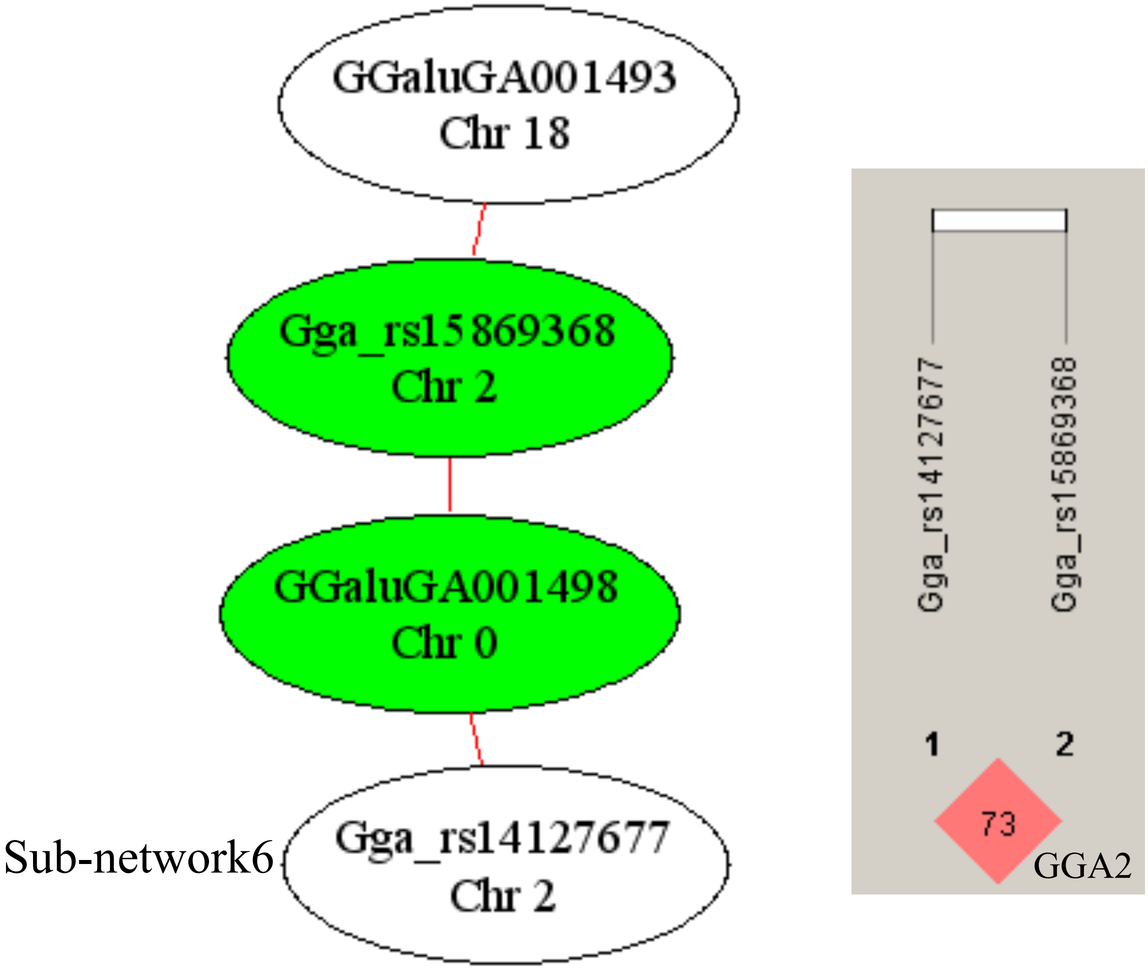

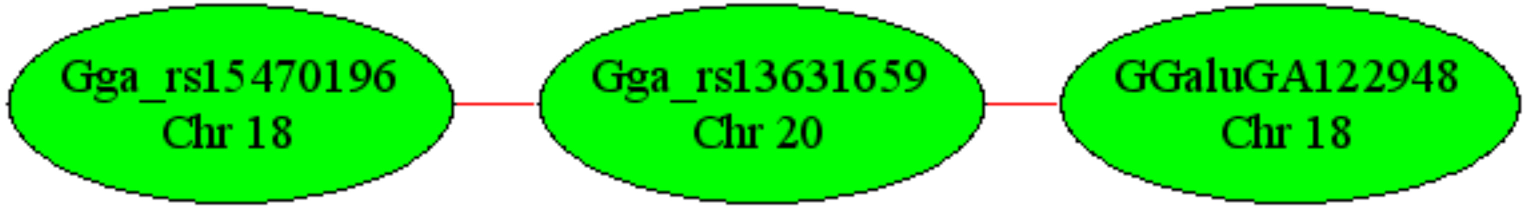

Sub-network7

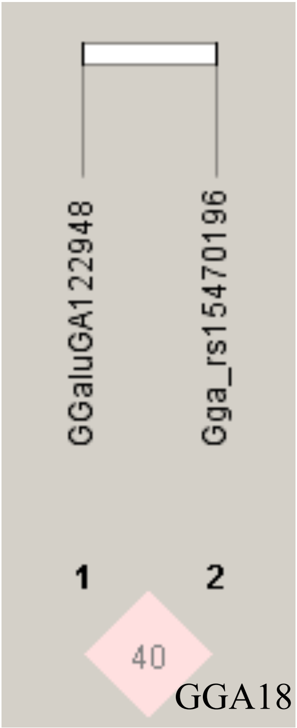

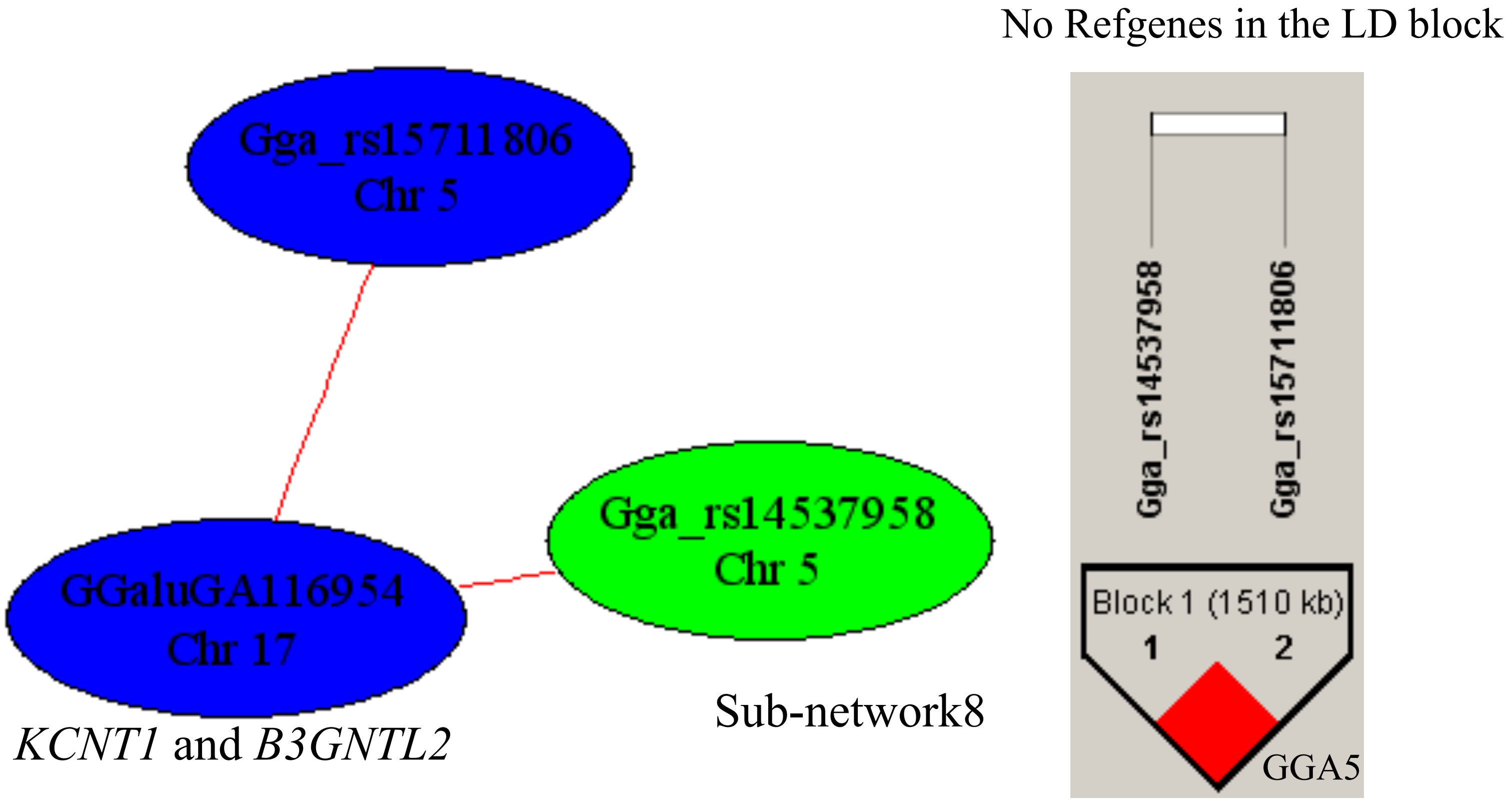
No Refgenes in the LD block
Gga_rs15711806
Chr 5
GGaluGA116954
Chr 17
Gga_rs14537958
Chr 5
KCNT1 and B3GNTL2
Sub-network8
Gga_rs14537958
Gga_rs15711806
Block 1 (1510 kb)
1
2
GGA5

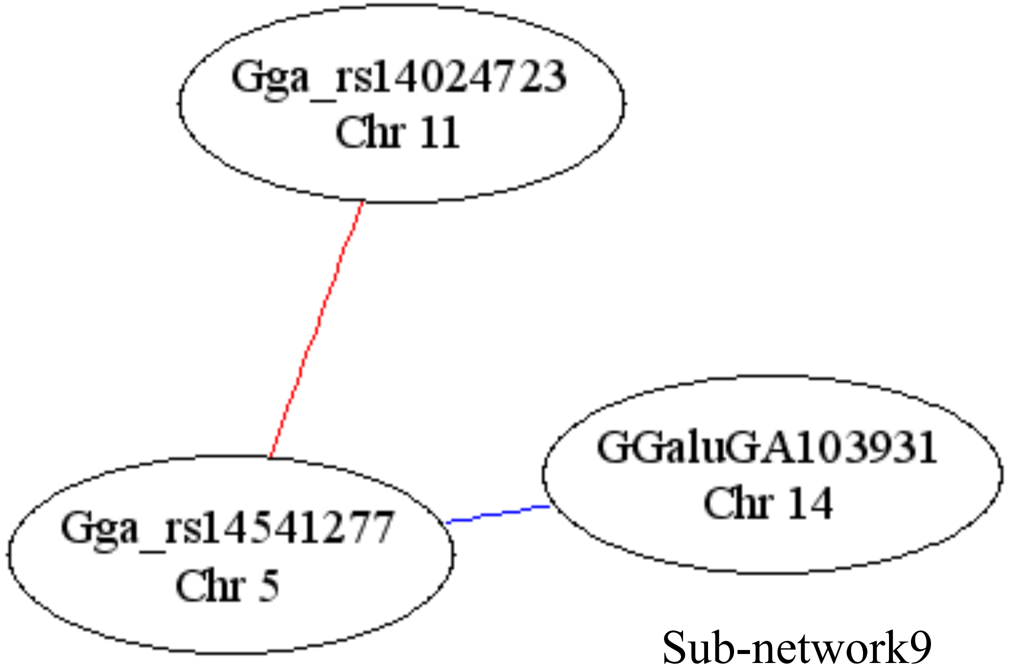

Sub-network9

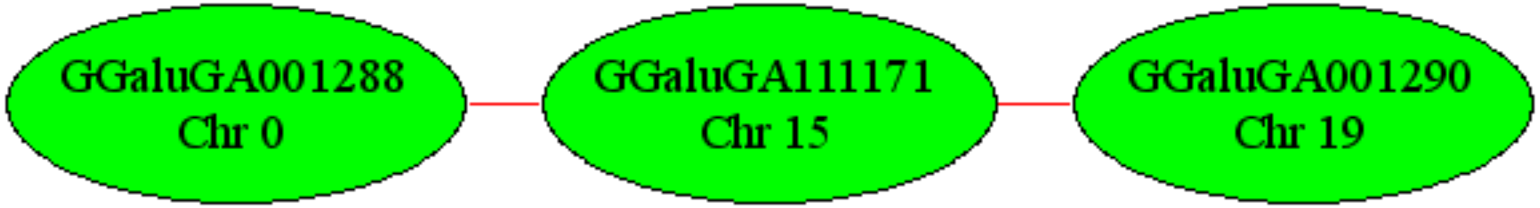

Sub-network10

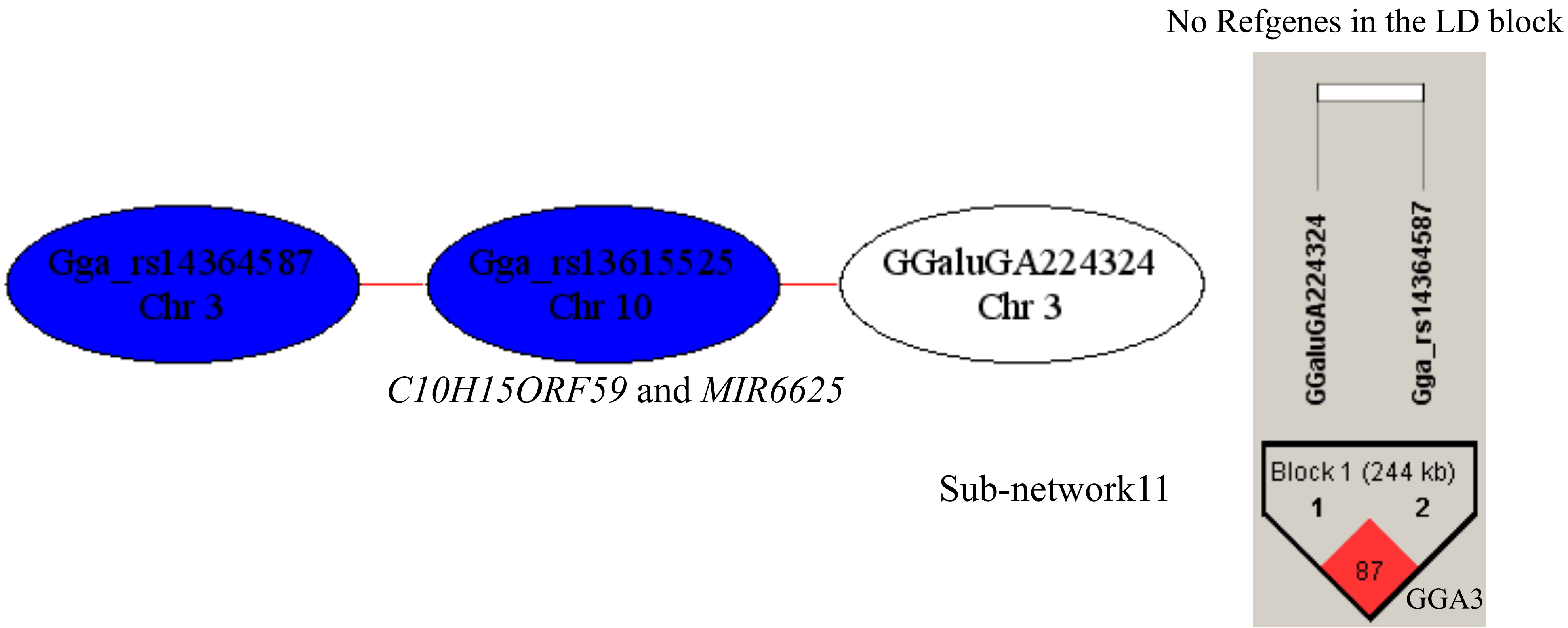
No Refgenes in the LD block
Gga_rs14364587
Chr 3
Gga_rs13615525
Chr 10
GGaluGA224324
Chr 3
C10H15ORF59 and MIR6625
GGaluGA224324
Gga_rs14364587
Sub-network11
Block 1 (244 kb)
1
2
87
GGA3
