# Supplementary material for: Identification of genome-wide SNP-SNP interactions associated with important traits in chicken
Source: BMC Genomics. 2017 Nov 21;18:892. doi: 10.1186/s12864-017-4252-y (PMC5698929; doi:10.1186/s12864-017-4252-y)
Supplement: Supplementary file 5 — The LD blocks of the SNPs on every chromosome in Sub-network1 for testis weight (TeW). This information was used to simply Sub-network1. (PDF 619 kb) [file 12864_2017_4252_MOESM5_ESM.pdf]

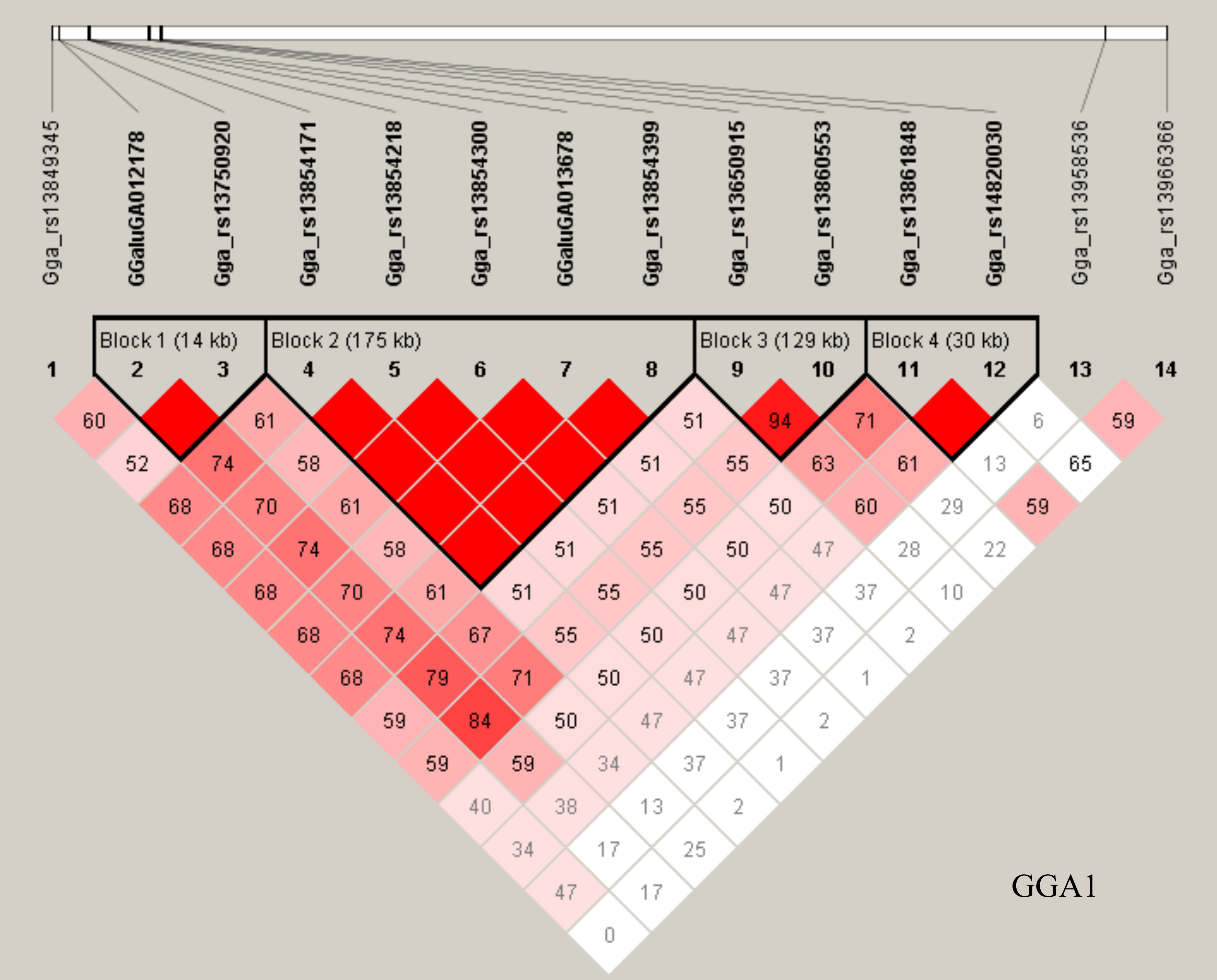
Gga_rs13849345
GGaluGA012178
Gga_rs13750920
Gga_rs13854171
Gga_rs13854218
Gga_rs13854300
GGaluGA013678
Gga_rs13854399
Gga_rs13650915
Gga_rs13860553
Gga_rs13861848
Gga_rs14820030
Gga_rs13958536
Gga_rs13966366
Block 1 (14 kb)
Block 2 (175 kb)
Block 3 (129 kb)
Block 4 (30 kb)
1 2 3 4 5 6 7 8 9 10 11 12 13 14

GGA1

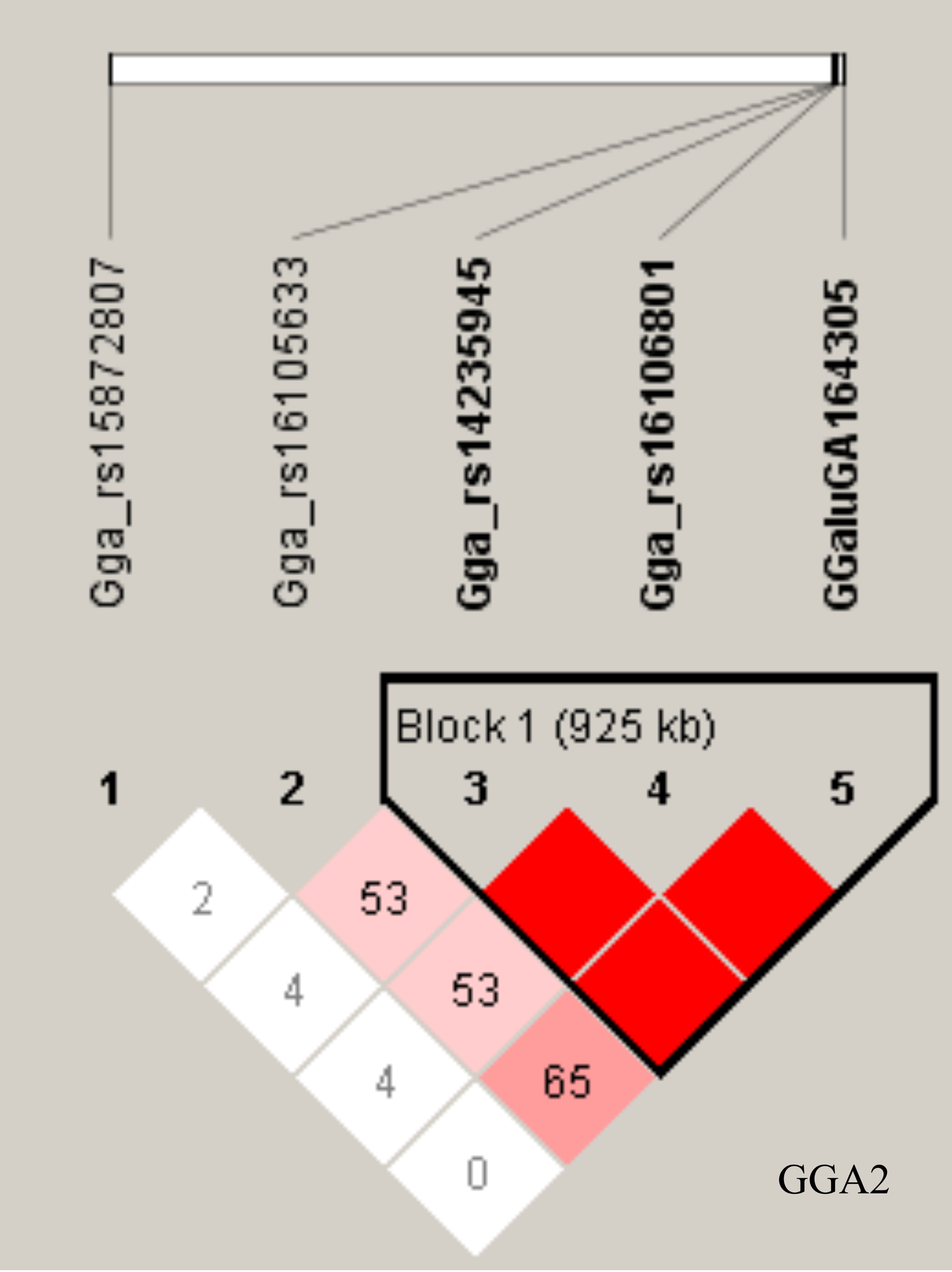
Gga_rs15872807
Gga_rs16105633
Gga_rs14235945
Gga_rs16106801
GGaluGA164305
Block 1 (925 kb)
1
2
3
4
5
2
53
4
53
4
65
0

GGA2

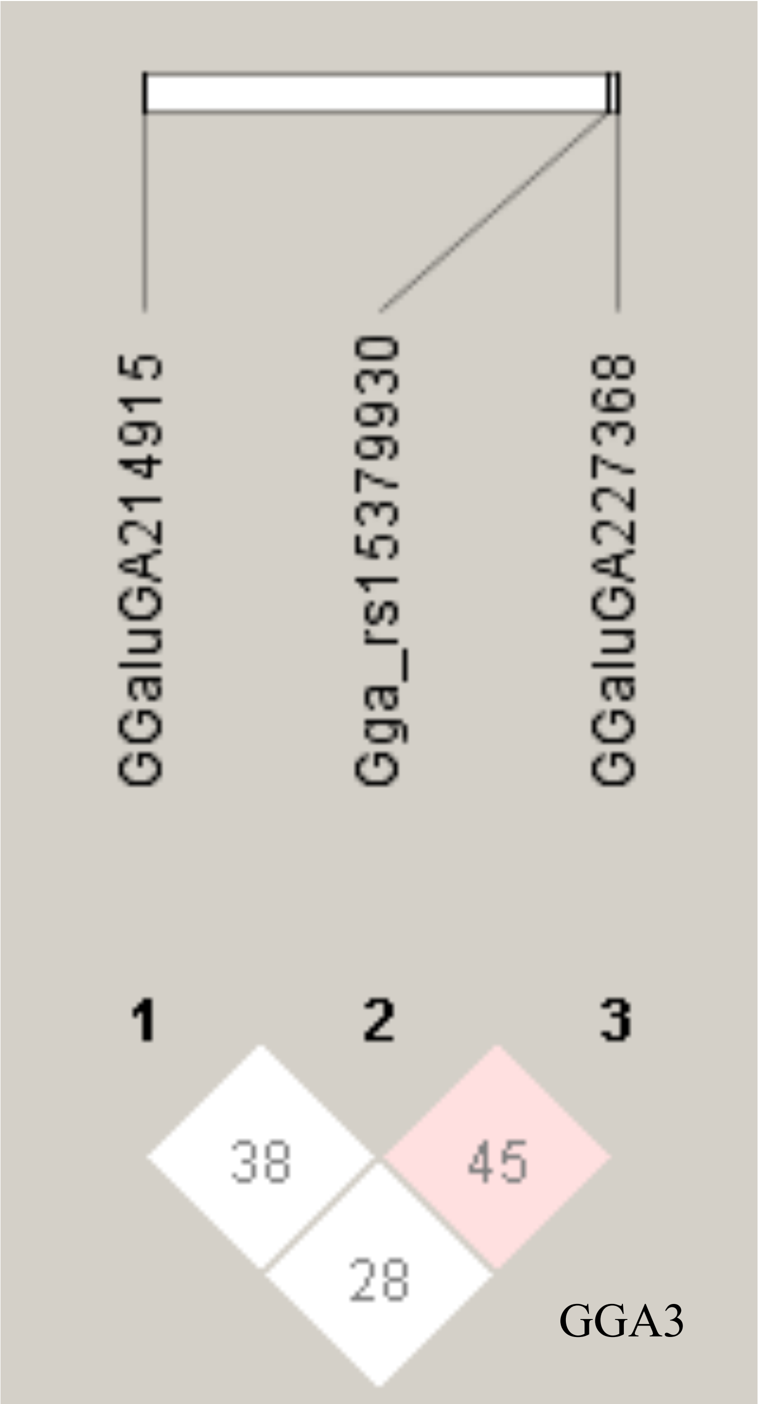

GGA3

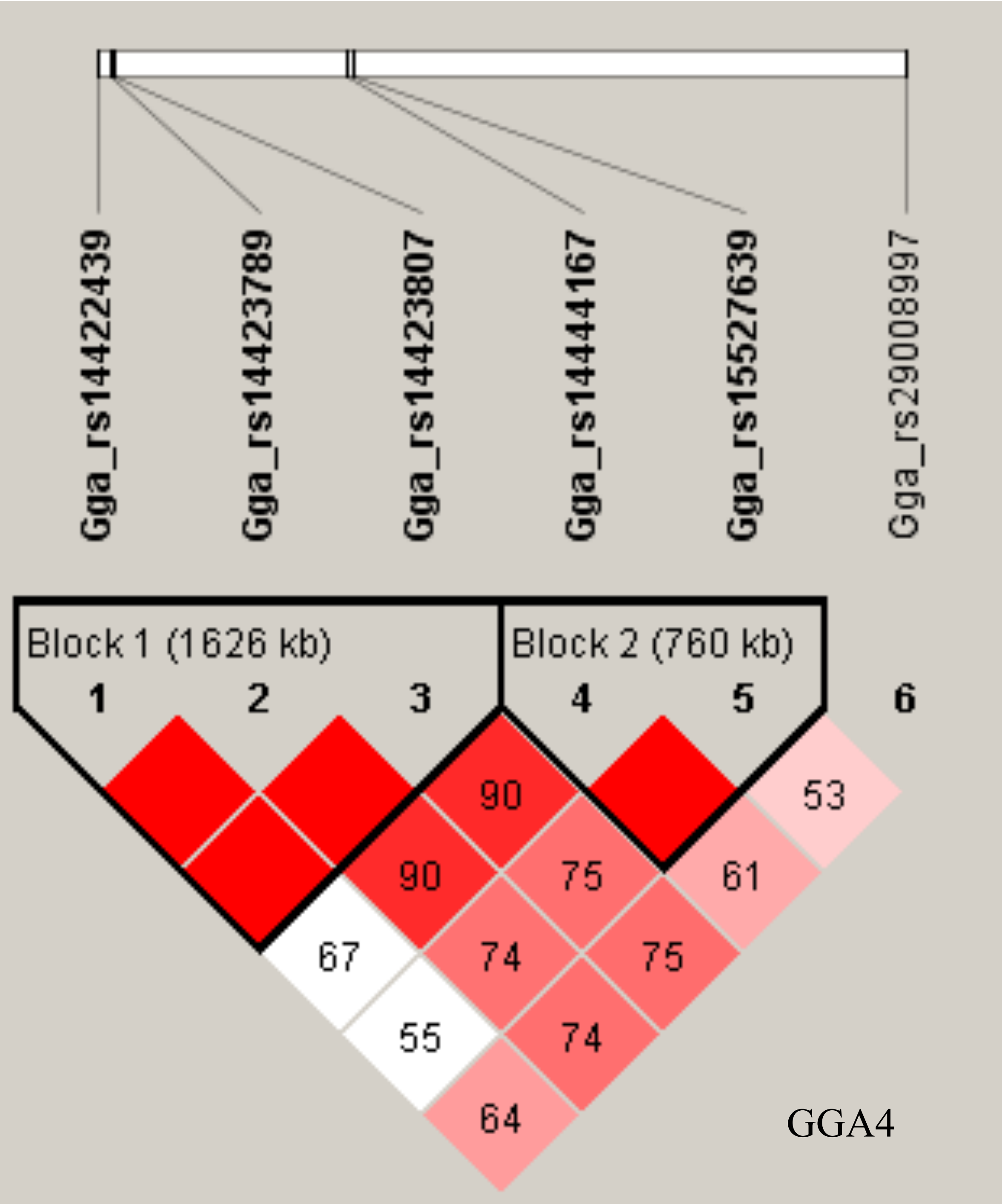

Gga_rs14422439
Gga_rs14423789
Gga_rs14423807
Gga_rs14444167
Gga_rs15527639
Gga_rs29008997
Block 1 (1626 kb)
Block 2 (760 kb)
1
2
3
4
5
6
90
53
90
75
61
67
74
75
55
74
64
GGA4

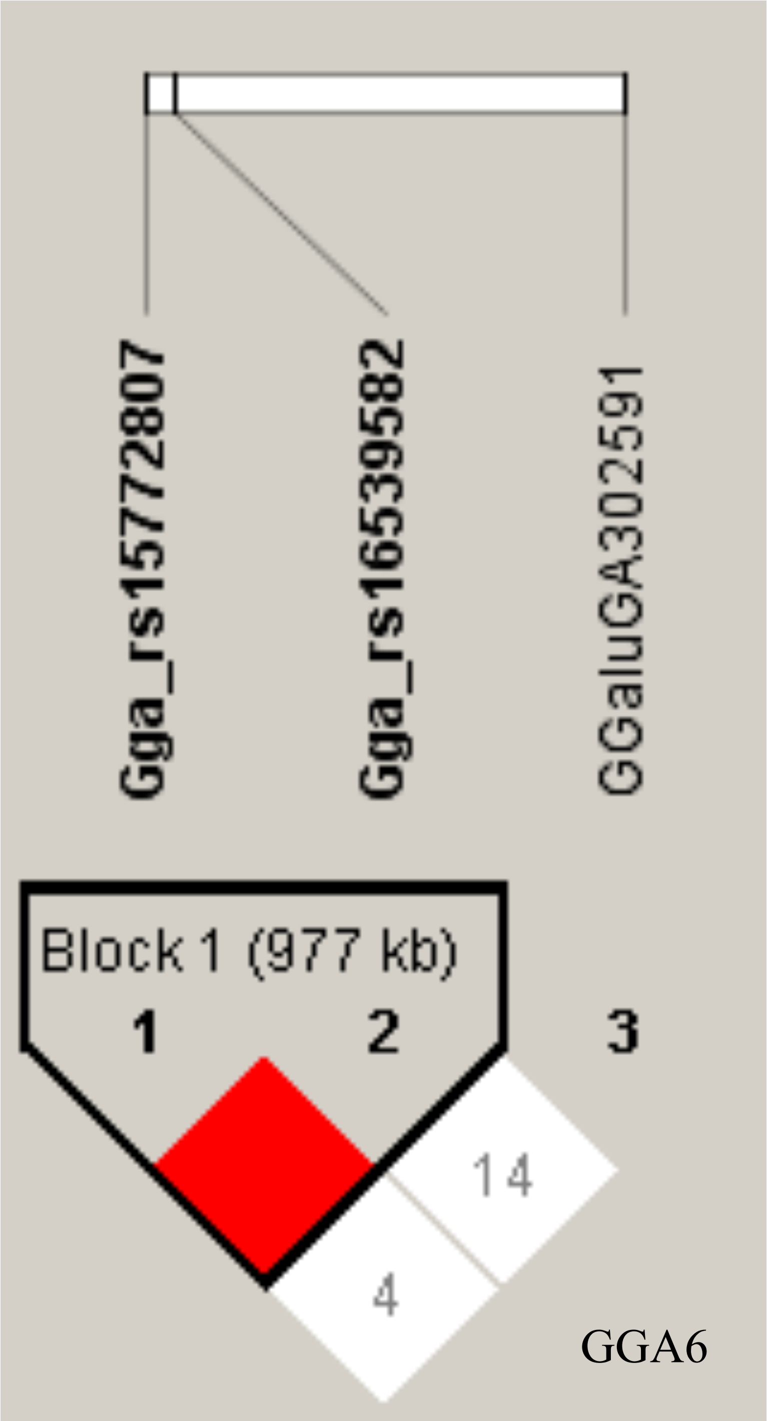

Gga_rs15772807
Gga_rs16539582
GGaluGA302591
Block 1 (977 kb)
1
2
3
14
4
GGA6

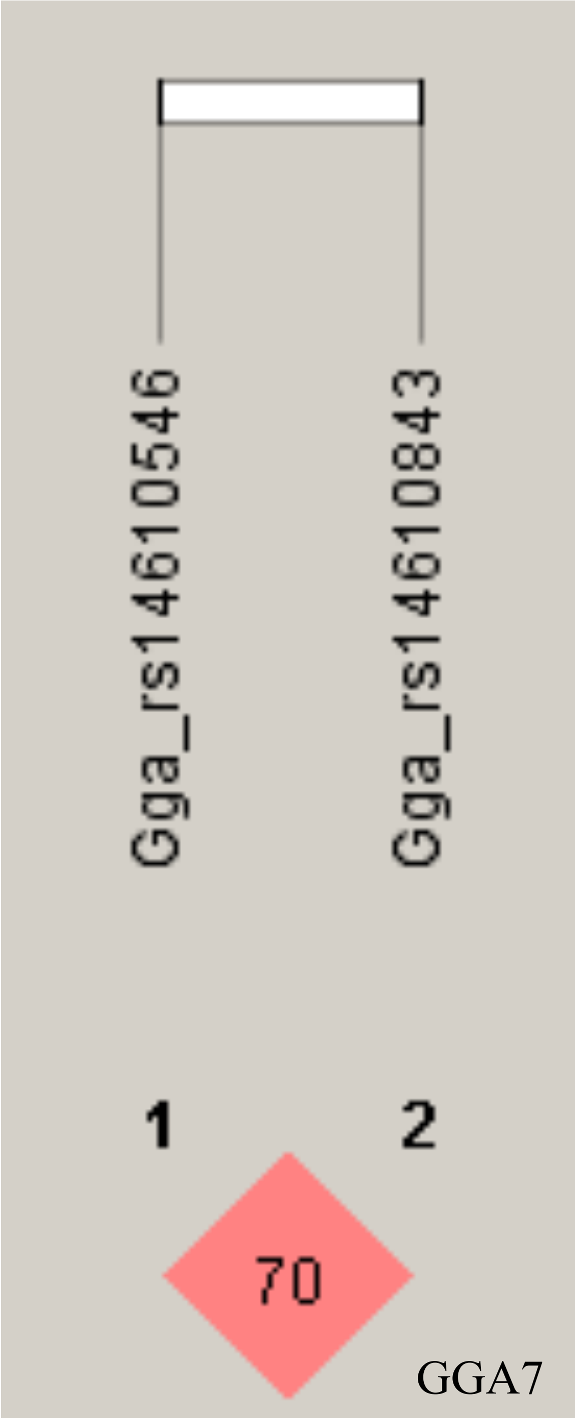

GGA7

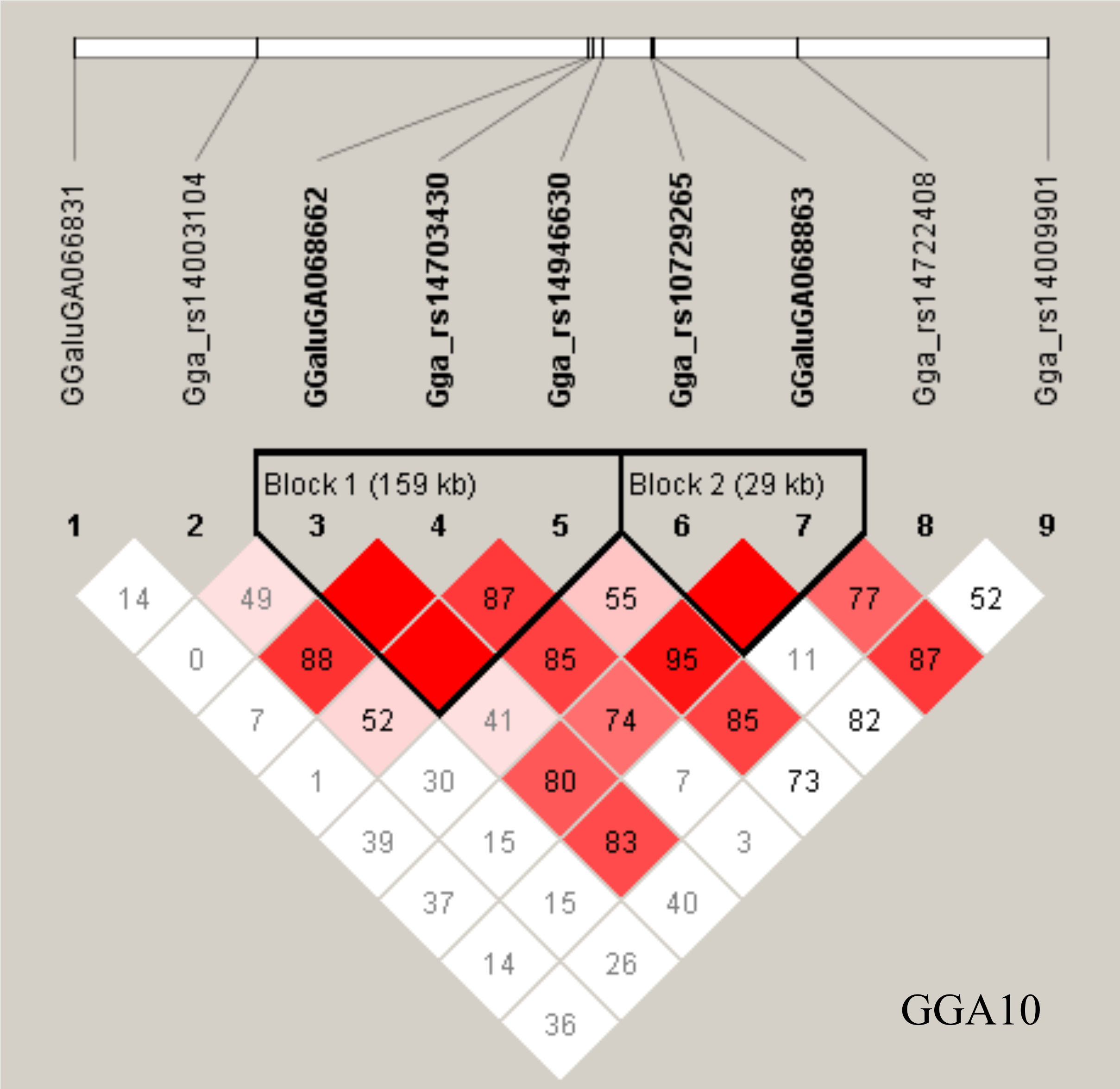

GGaluGA066831
Gga_rs14003104
GGaluGA068662
Gga_rs14703430
Gga_rs14946630
Gga_rs10729265
GGaluGA068863
Gga_rs14722408
Gga_rs14009901
Block 1 (159 kb)
Block 2 (29 kb)
1
2
3
4
5
6
7
8
9
GGA10

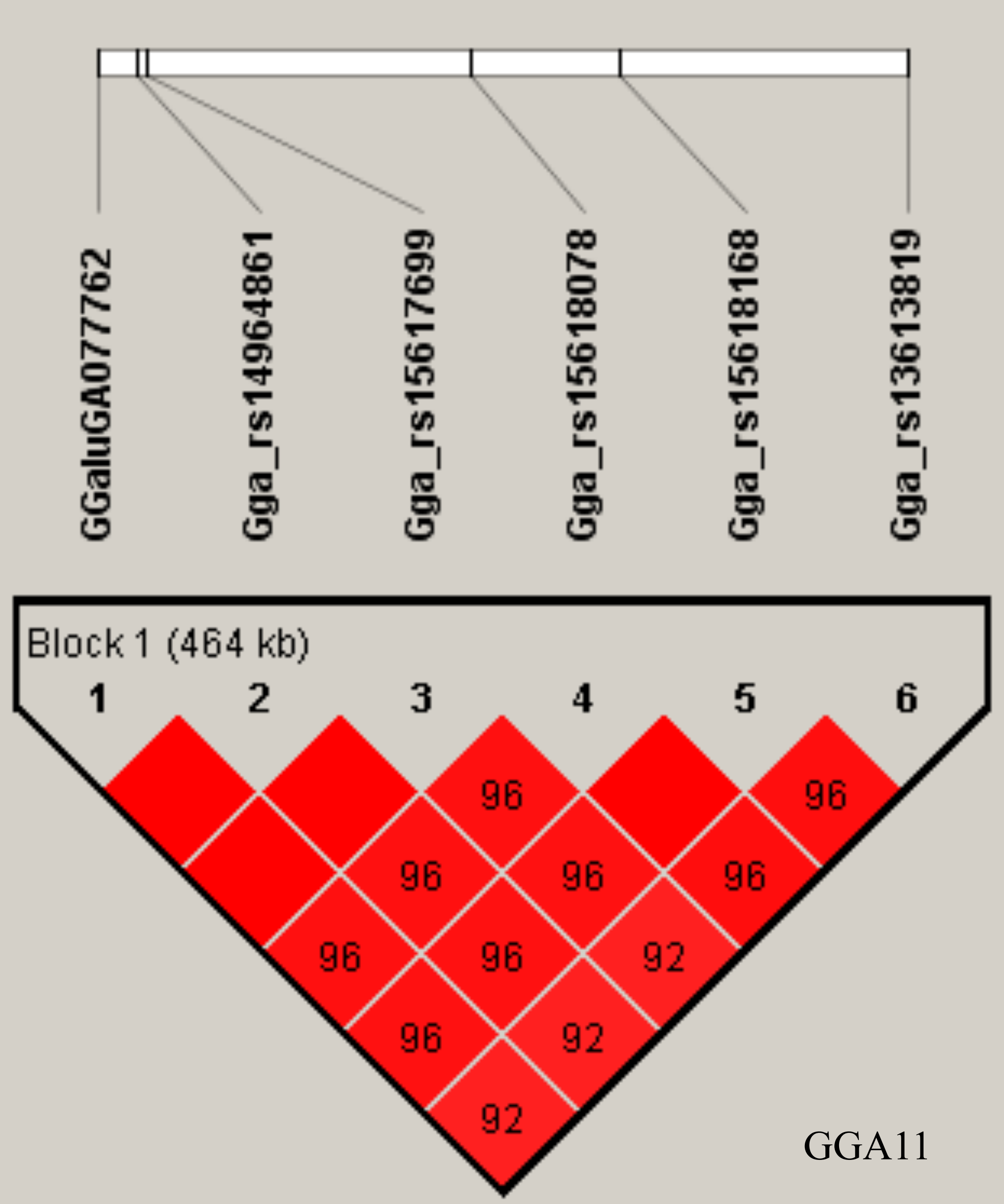
GGaluGA077762
Gga_rs14964861
Gga_rs15617699
Gga_rs15618078
Gga_rs15618168
Gga_rs13613819
Block 1 (464 kb)
1
2
3
4
5
6
96
96
96
96
96
96
96
96
92
92
92
GGA11

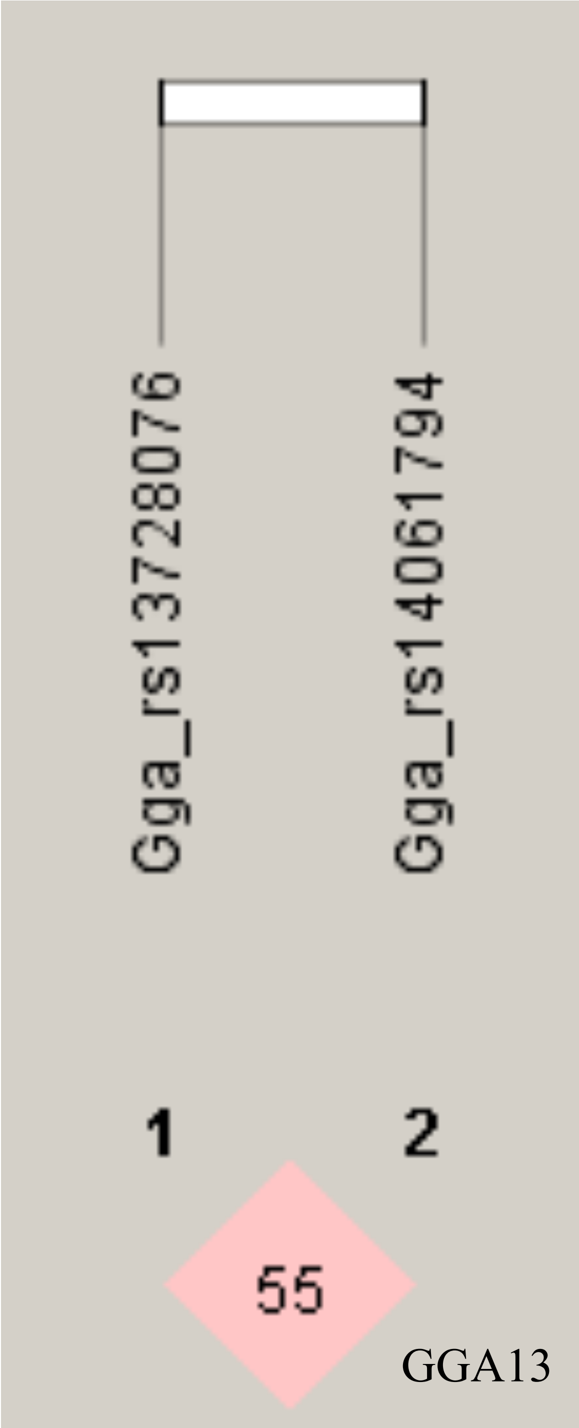
Gga_rs13728076
Gga_rs14061794
1
2
55
GGA13

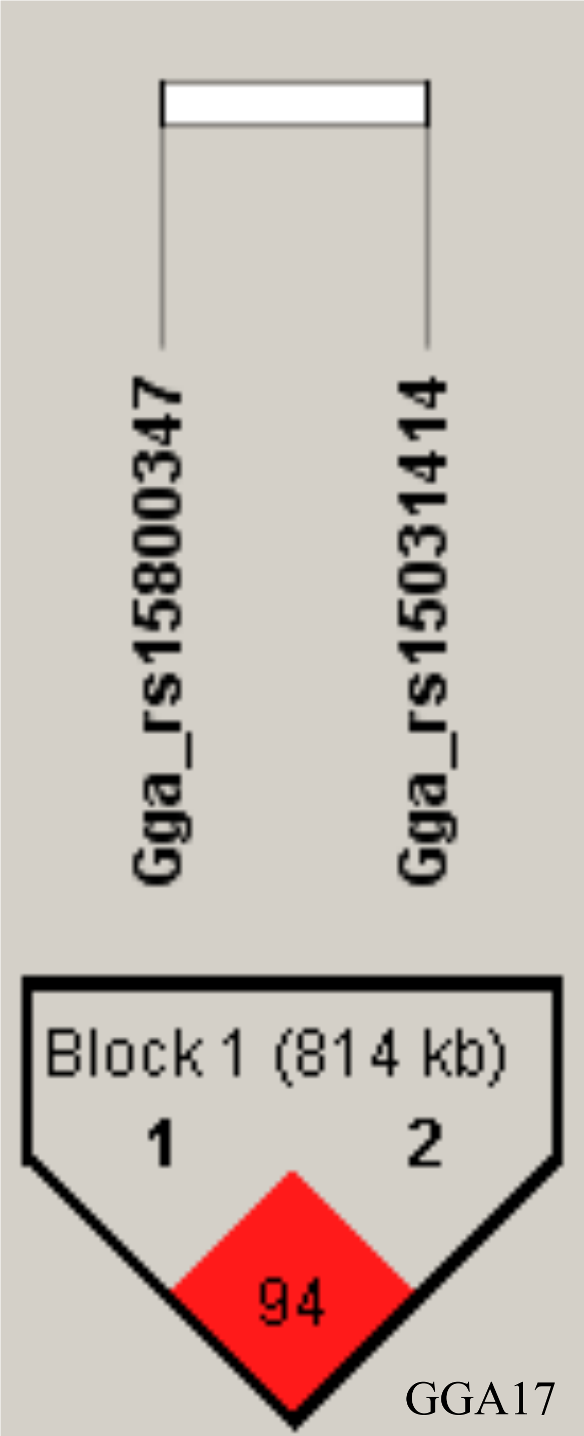
Gga_rs15800347
Gga_rs15031414
Block 1 (814 kb)
1
2
94
GGA17

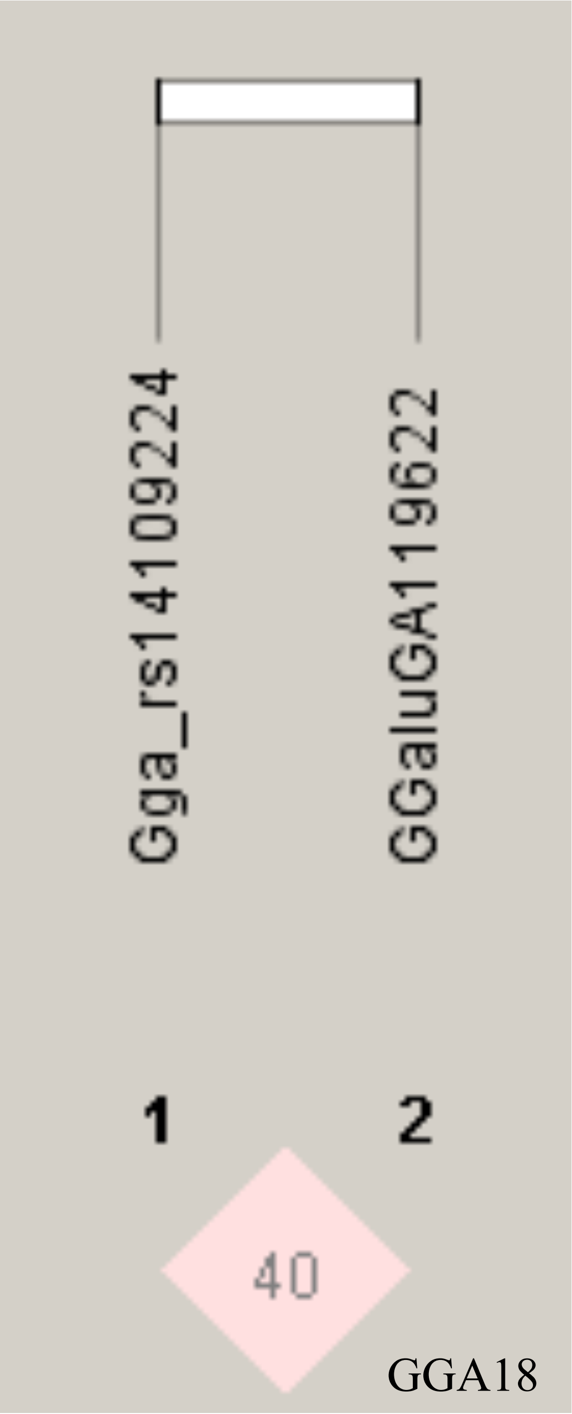
Gga_rs14109224
GGaluGA119622
1
2
40
GGA18

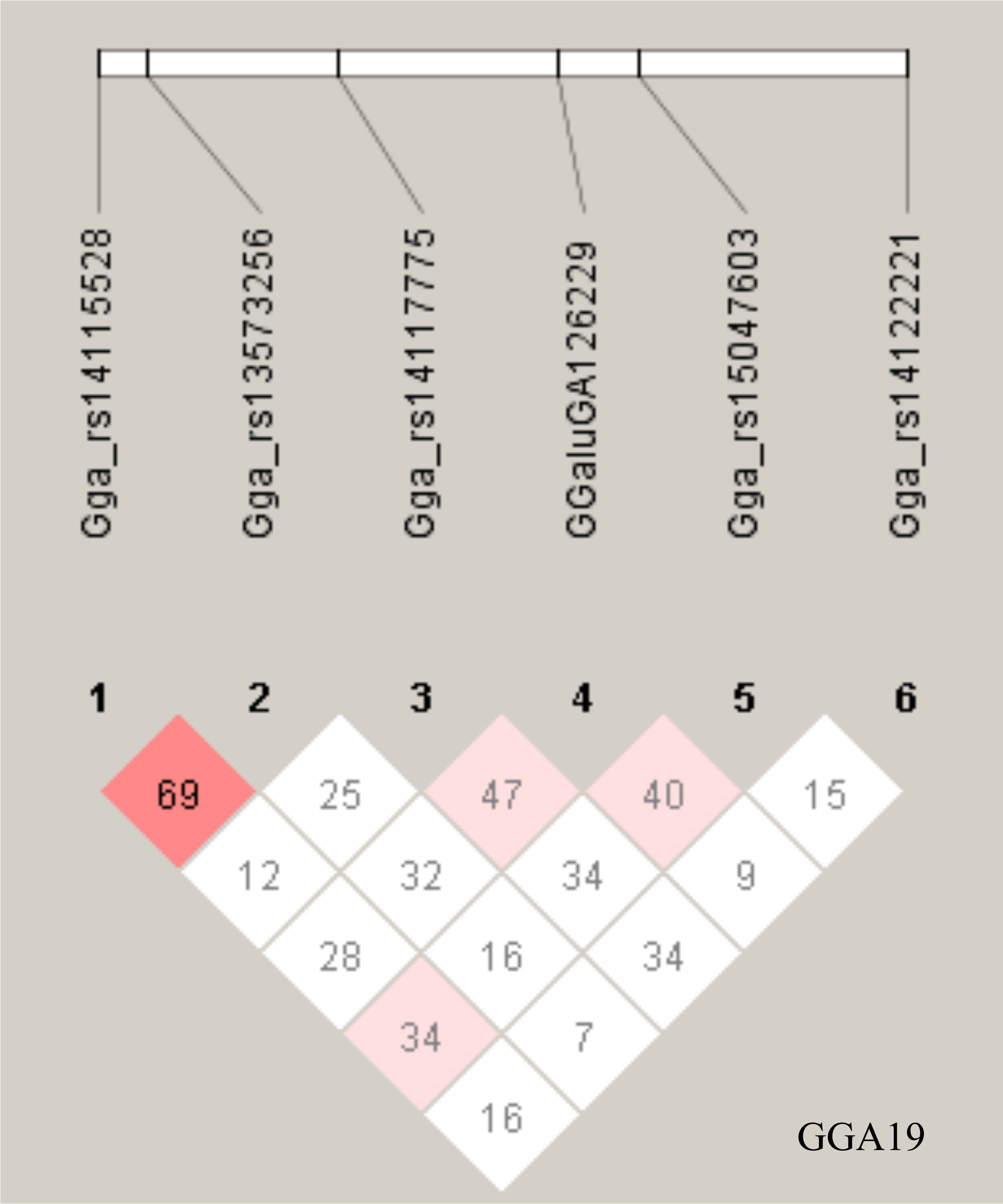
Gga_rs14115528
Gga_rs13573256
Gga_rs14117775
GGaluGA126229
Gga_rs15047603
Gga_rs14122221
1
2
3
4
5
6
69
25
47
40
15
12
32
34
9
28
16
34
34
7
16
GGA19

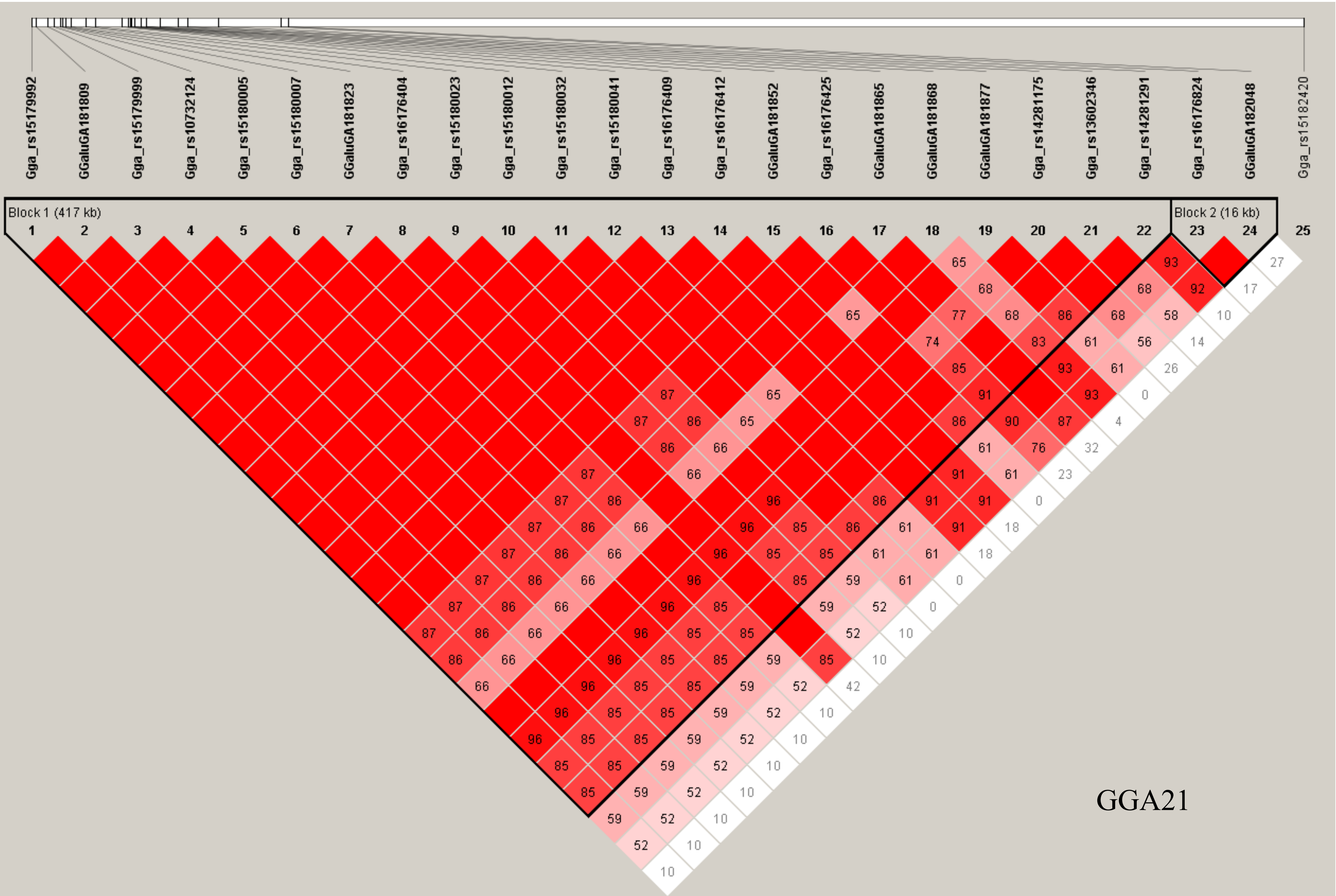

Gga_rs15179992
GGaluGA181809
Gga_rs15179999
Gga_rs10732124
Gga_rs15180005
Gga_rs15180007
GGaluGA181823
Gga_rs16176404
Gga_rs15180023
Gga_rs15180012
Gga_rs15180032
Gga_rs15180041
Gga_rs16176409
Gga_rs16176412
GGaluGA181852
Gga_rs16176425
GGaluGA181865
GGaluGA181868
GGaluGA181877
Gga_rs14281175
Gga_rs13602346
Gga_rs14281291
Gga_rs16176824
GGaluGA182048
Gga_rs15182420
Block 1 (417 kb)
Block 2 (16 kb)
1 2 3 4 5 6 7 8 9 10 11 12 13 14 15 16 17 18 19 20 21 22 23 24 25
GGA21

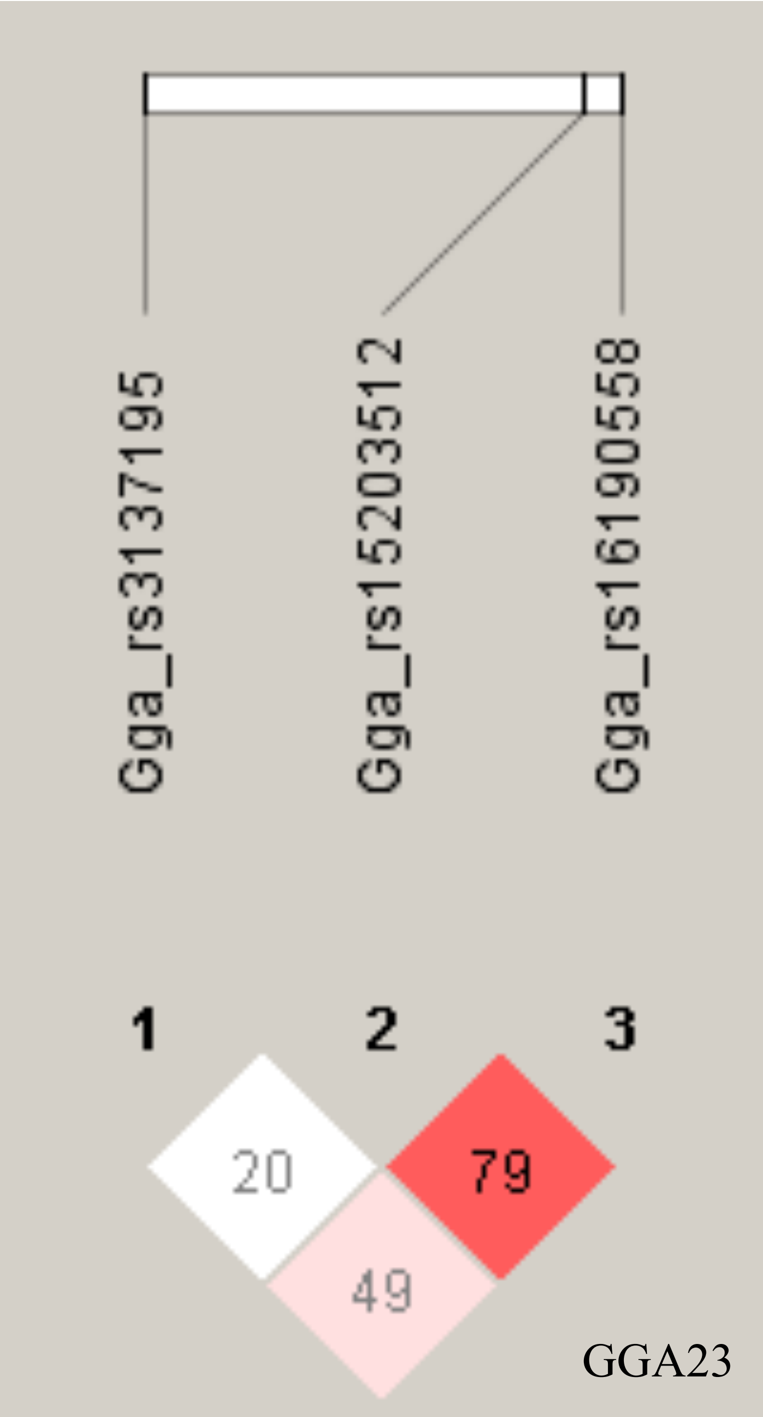
Gga_rs31371195
Gga_rs15203512
Gga_rs16190558
1
2
3
20
79
49
GGA23

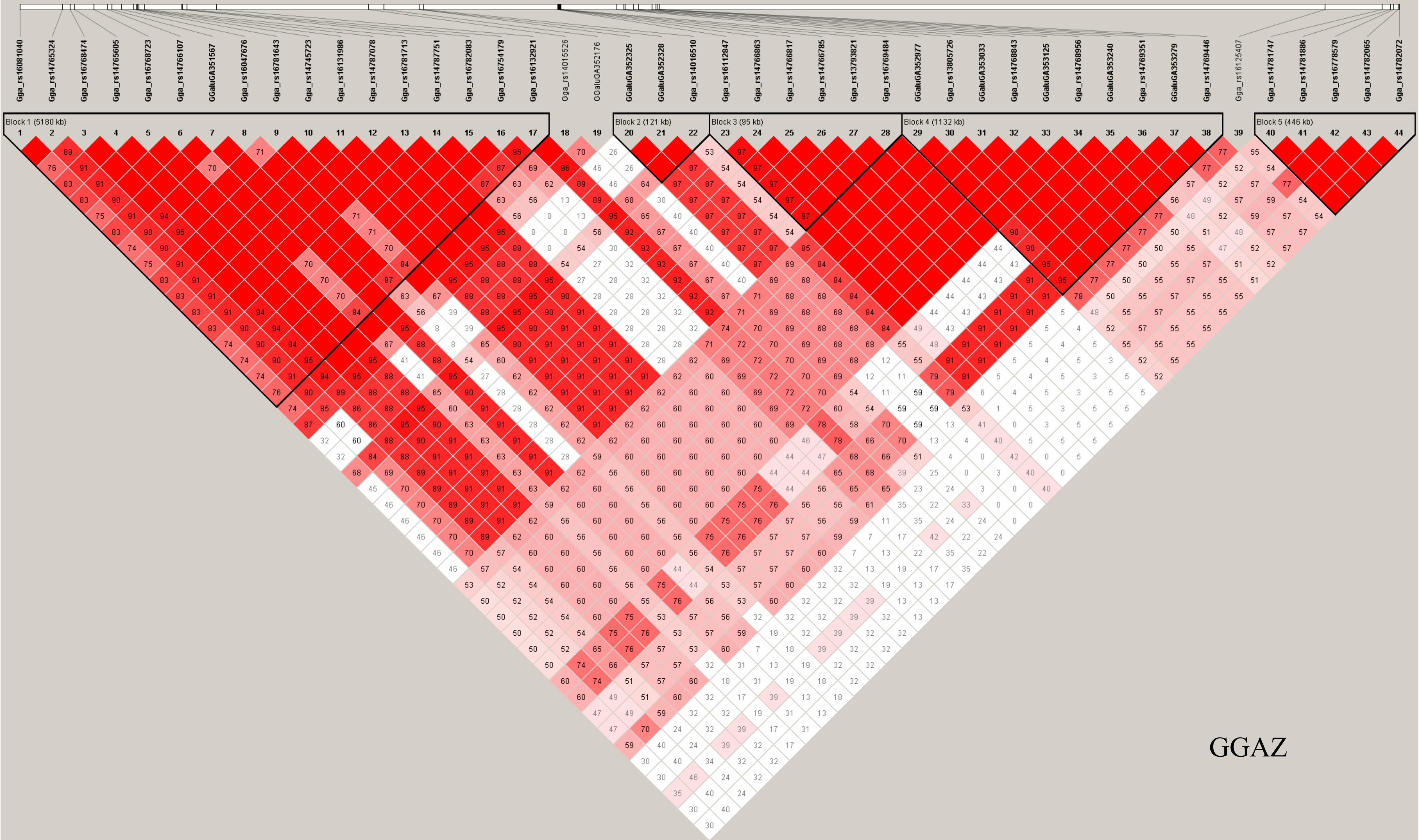
Gga_rs16081040
Gga_rs14765324
Gga_rs16768474
Gga_rs14765605
Gga_rs16768723
Gga_rs14766107
GGaluGA351567
Gga_rs16047676
Gga_rs16781643
Gga_rs14745723
Gga_rs16131986
Gga_rs14787078
Gga_rs16781713
Gga_rs14787751
Gga_rs16782083
Gga_rs16754179
Gga_rs16132921
Gga_rs14015526
GGaluGA352176
GGaluGA352325
GGaluGA352328
Gga_rs14016510
Gga_rs16112847
Gga_rs14766863
Gga_rs14766817
Gga_rs14766785
Gga_rs13793821
Gga_rs16769484
GGaluGA352977
Gga_rs13805726
GGaluGA353033
Gga_rs14768843
GGaluGA353125
Gga_rs14768956
GGaluGA353240
Gga_rs14769351
GGaluGA353279
Gga_rs14769446
Gga_rs16125407
Gga_rs14781747
Gga_rs14781886
Gga_rs16778579
Gga_rs14782065
Gga_rs14782072
Block 1 (5180 kb)
Block 2 (121 kb)
Block 3 (95 kb)
Block 4 (1132 kb)
Block 5 (446 kb)
GGAZ
